# Providing mental health first aid in the workplace: a Delphi consensus study

**DOI:** 10.1186/s40359-016-0148-x

**Published:** 2016-08-02

**Authors:** Nataly Bovopoulos, Anthony F. Jorm, Kathy S. Bond, Anthony D. LaMontagne, Nicola J. Reavley, Claire M. Kelly, Betty A. Kitchener, Angela Martin

**Affiliations:** 1 Centre for Mental Health, Melbourne School of Population and Global Health, University of Melbourne, 207 Bouverie St, Parkville, VIC 3010 Australia; 2 Mental Health First Aid Australia, Level 6, 369 Royal Parade, Parkville, VIC 3052 Australia; 3 Centre for Population Health Research, School of Health & Social Development, Deakin University, Geelong, VIC 3220 Australia; 4 Centre for Health Equity, Melbourne School of Population & Global Health, University of Melbourne, Level 207 Bouverie St, Parkville, VIC 3010 Australia; 5 School of Psychology, Deakin University, 1 Gheringhap St, Geelong, VIC 3220 Australia; 6 Tasmanian School of Business and Economics, University of Tasmania, Private Bag 84, Hobart, TAS 7001 Australia

**Keywords:** Mental health first aid, Workplace, Delphi method, Workplace guidelines

## Abstract

**Background:**

Mental health problems are common in the workplace, but workers affected by such problems are not always well supported by managers and co-workers. Guidelines exist for the public on how to provide mental health first aid, but not specifically on how to tailor one’s approach if the person of concern is a co-worker or employee. A Delphi consensus study was carried out to develop guidelines on additional considerations required when offering mental health first aid in a workplace context.

**Methods:**

A systematic search of websites, books and journal articles was conducted to develop a questionnaire with 246 items containing actions that someone may use to offer mental health first aid to a co-worker or employee. Three panels of experts from English-speaking countries were recruited (23 consumers, 26 managers and 38 workplace mental health professionals), who independently rated the items over three rounds for inclusion in the guidelines.

**Results:**

The retention rate of the expert panellists across the three rounds was 61.7 %. Of the 246 items, 201 items were agreed to be important or very important by at least 80 % of panellists. These 201 endorsed items included actions on how to approach and offer support to a co-worker, and additional considerations where the person assisting is a supervisor or manager, or is assisting in crisis situations such as acute distress.

**Conclusions:**

The guidelines outline strategies for a worker to use when they are concerned about the mental health of a co-worker or employee. They will be used to inform future tailoring of Mental Health First Aid training when it is delivered in workplace settings and could influence organisational policies and procedures.

**Electronic supplementary material:**

The online version of this article (doi:10.1186/s40359-016-0148-x) contains supplementary material, which is available to authorized users.

## Background

Depression and anxiety disorders are highly prevalent mental illnesses and are among the leading causes of disability worldwide [1, 2]. Mental illnesses can significantly affect workplace productivity due to absenteeism and presenteeism (working whilst unwell and not meeting expected standards of productivity) [3]. The majority of full-time employees experiencing common mental illness do not receive treatment, or delay seeking treatment [4, 5]. The longer treatment is delayed, the more likely a person will have a long-term sickness absence from work [6]. There is also strong evidence of a positive relationship between the duration of depression and the severity of work disability experienced [7].

There are a number of workplace barriers to help-seeking, including lack of recognition, stigmatising attitudes and discrimination. For example, in a comprehensive study with 60,556 Australian workers, 31 % of full-time workers with high psychological distress did not recognise that they had a mental health problem and consequently had not sought any help [4]. Addressing workers’ lack of recognition for needed help, attitudes and structural barriers can significantly decrease work productivity losses, with the largest decrease associated with improving recognition [8]. A recent study across 35 countries found that people with depression had significant expected and actual experiences of discrimination in the workplace [9]. Concerns about being treated differently by co-workers and supervisors, being seen as less competent and possible job loss have been cited as barriers to disclosing a mental illness at work [10]. Managers have been found to be more critical of an employee’s job performance if they are seen to be experiencing burnout or depression compared to a physical illness like thyroid disease [11]. Poor support from managers in turn has been found to double the risk of long-term sickness absence related to mental illness [12].

The impact of mental illness in the workplace could be reduced by facilitating early intervention that encourages help-seeking and supports recovery through reasonable work accommodation [13]. A factor that can facilitate professional help-seeking is that someone else suggests it, such as a co-worker or manager [14, 15]. Mental Health First Aid (MHFA) training is one way to promote early help-seeking, by equipping participants with the knowledge and skills to provide the initial help to someone developing a mental health problem or experiencing a mental health crisis before appropriate professional help is found [16]. MHFA courses have been well evaluated and consistently demonstrate an association with improved knowledge, decreased stigmatizing attitudes and increased helping behaviours [17].

The MHFA curriculum is drawn from guidelines developed using the expert consensus of mental health professionals, consumers and carer advocates from developed English-speaking countries [18–25]. Guidelines for providing mental health first aid in particular settings have also been developed to inform MHFA course adaptations. For example, guidelines on how to provide culturally appropriate mental health first aid to Aboriginal and Torres Strait Islander adults and adolescents have informed the Aboriginal and Torres Strait Islander MHFA Course [26, 27]. Though guidelines exist on how to help someone return to work after depression and anxiety and how organisations can help prevent mental illness, there are no guidelines on how to offer help in a workplace setting that can be used to inform a course for the workplace [28, 29].

The Standard MHFA course was developed for the general community, akin to physical first aid, and has been delivered and shown to be effective in workplace settings in three randomised controlled trials in Australia [30], Denmark [31] and Sweden [32]. However, none of the courses evaluated in these studies aimed to improve the helping responses specifically of workers toward their peers in the workplace. Rather, these trials focused on improving helping responses towards any adult, suggesting that the use of a workplace sample was more for convenience. A survey of Australian MHFA Instructors exploring their experiences of delivering training to workplaces indicated a demand to further tailor the content towards co-workers helping one another [33]. In addition, a mental health literacy intervention for university staff which included MHFA training, recommended that future MHFA research consider tailoring the course further and include strategies that target work-related risk and protective factors [34]. The additional considerations of delivering mental health first aid in a workplace that have not been covered within existing guidelines or curricula include the workplace relationship between the mental health first aider and the recipient, the potential overlap and conflicts with performance management and concerns around workplace discrimination on the basis of mental health status.

The aim of this study was to establish guidelines on how someone should apply mental health first aid to a co-worker or employee developing or experiencing a common mental health problem (symptoms of depression, anxiety and substance use problems which may meet criteria for a diagnosis of a mental illness). The guidelines will be used to inform a tailored MHFA course directed at improving workers’ helping response to their co-workers. These guidelines are intended to complement two other workplace-related guidelines already developed, which both used the Delphi methodology - preventing common mental illnesses in workplaces [28] and helping an employee return-to-work during recovery from a common mental illness [29].

## Methods

The Delphi expert consensus method was used. This method establishes the consensus of expert panels on a particular topic and is often used for the development of guidelines [35]. As used here, the expert panellists rated a series of statements about potential mental health first aid actions in workplace settings, which were generated from a comprehensive search of the literature.

### Panel formation

Consumer advocates, managers and workplace mental health professionals considered to have expert knowledge in the field of workplace mental health were recruited from five English-speaking developed countries to participate in the study through the distribution of an advertisement with a link to the plain language statement. See Table 1 for the inclusion criteria. The study aimed to recruit 30 people in each panel. This panel size is within the typical Delphi panel size of 15–60 experts [36]. A panel size of 23 has been found to yield stable results in a simulation study [37].

**Table 1:** Inclusion criteria

Panel	Criteria
Consumer	Be 18 years or older, AND Live in Australia, Canada, Ireland, New Zealand, United Kingdom or the United States, AND Have lived experience of mental health problems whilst working, AND Have experience in an advocacy role, AND Symptoms of mental health problems are currently well managed.
Manager	Be 18 years or older, AND Live in Australia, Canada, Ireland, New Zealand, United Kingdom or the United States, AND Have a minimum of 5 years’ management experience, AND Training in mental health, OR Practical experience in supervising employees with mental health problems.
Workplace Mental Health Professional	Be 18 years or older, AND Live in Australia, Canada, Ireland, New Zealand, United Kingdom or the United States, AND Have a minimum of 5 years’ experience in their profession, AND Clinical, policy, program and/or research experience regarding mental health issues in the workplace.

Consumer advocates were recruited via consumer advocacy groups and organisations focused on mental health. Managers were recruited through employer representative organisations (e.g. Chambers of Commerce) and workplace mental health groups (e.g. the HeadsUp Australian campaign LinkedIn group). Workplace mental health professionals were recruited through direct contact with the primary author. The study did not aim to obtain representative samples of experts, as the Delphi method aims to find panel members who can draw on a diverse range of relevant expertise [35].

### Literature search and questionnaire development

A comprehensive literature search, using key terms, was conducted of websites, journal articles, books, booklets and training manuals. This search aimed to find relevant statements on how a worker can provide mental health first aid to a co-worker or employee developing early signs or symptoms of a common mental illness.

The key search terms used were (work* OR manager OR staff OR employee OR employer) AND (mental health OR mental illness OR mental disorder OR depression OR anxiety OR substance use OR alcohol OR drug OR distress OR stress) AND (first aid OR help OR assistance OR support OR early intervention). See Table 2 for details.

**Table 2:** Search strategies and results

Search source	Search terms	Search results	Examples
www.google.com.au www.google.co.uk www.google.ca www.google.com	(Work* OR manager OR staff OR employee OR employer) AND (mental health OR mental illness OR mental disorder OR depression OR anxiety OR substance use OR alcohol OR drug OR distress OR stress) AND (first aid OR help OR assistance OR support OR early intervention)	266 websites	http://nceta.flinders.edu.au/files/5512/6465/9927/EN149_NCETA%20Booklet%2012006.pdf www.headsup.org.au/supporting-others-in-the-workplace/having-a-conversation
books.google.com amazon.com	(Work* OR manager OR staff OR employee OR employer) AND (mental health OR mental illness OR mental disorder OR depression OR anxiety OR substance use OR alcohol OR drug OR distress OR stress) AND (first aid OR help OR assistance OR support OR early intervention)	9 Books	Eyers & Parker [45]
PsychInfo PubMed	(Work* OR manager OR staff OR employee OR employer) AND (mental health OR mental illness OR mental disorder OR depression OR anxiety OR substance use OR alcohol OR drug OR distress OR stress) AND (first aid OR help OR assistance OR support OR early intervention) in the abstract or text of the article.	35 Journal Articles	Joyce [43] Moll [44]

*Use a wildcard search in order to include terms such as workplace or worker

These search terms were used to find relevant English-language websites, books and journal articles. Relevant websites were found via google.com.au, google.com, google.com.ca and google.com.uk. The first 50 hits were retrieved and those that appeared relevant were reviewed by the researcher in order to find helping statements. Relevant books were searched via books.google.com.au and amazon.com. The first 50 hits were reviewed from the top selling books. Relevant journal articles published in the last 10 years (2003–2013) were found via PsycInfo and PubMed databases, searching via title or abstract.

The statements identified by the first author were then categorised thematically in a spreadsheet and distilled into statements that were instructional, unambiguous and contained one idea per statement. A total of 310 resources were used to develop the Round 1 survey (see Table 2).

In consultation with a working group consisting of four staff from Mental Health First Aid Australia, and three workplace mental health researchers from the University of Melbourne and Deakin University (the authors of this paper), the statements were reworded for consistency and to avoid repetition, whilst remaining true to the meaning of the original text. The working group of researchers met on several occasions to finalise the statements, which resulted in the Round 1 questionnaire organised thematically into 11 sections: General Awareness, Training, Approach, Communication, Performance, Follow-up, Distress, Support, Reasonable Adjustments, Disclosure and Substance Use Problems and Intoxication. In total, the working group met for more than 60 h to finalise the survey, process feedback and draft the guidelines. See Additional file 1: for a copy of the surveys.

### Data collection and analysis

Data was collected in three survey rounds administered online using SurveyMonkey between August 2015 and February 2016. Participants were invited to complete the Round 1 questionnaire, which asked them to rate whether certain actions should be included in guidelines on how a worker or manager should provide mental health first aid to a co-worker or an employee. Panellists rated each action to indicate its importance in being included in the guidelines on the following 5-point scale: ‘essential’, ‘important’, ‘don’t know/depends’, ‘unimportant’ or ‘should not be included’. During Round 1, participants were able to submit additional comments or suggestions to be included as actions to be rated by the panellists in the Round 2 survey. The qualitative data were collected by asking in each survey section, “Do you have any additional statements that you would like to add in this section? Please write your suggestions in the box provided.”

After panel members completed a survey round, the data was analysed using a spreadsheet to calculate the endorsement percentages by participants for each statement. The statements were categorised as follows:

1. Endorsed. The item received an ‘essential’ or ‘important’ rating from 80–100 % of each panel.
2. Re-rate. The item received an ‘essential’ or ‘important’ rating from 70–79 % of each panel, or an ‘essential’ or ‘important’ rating from 70–79 % of members from at least one panel and above 80 % from the other panels.
3. Rejected. The item did not fall into either the endorsed or re-rate categories.

The working group thematically analysed participants’ comments and translated these into new actionable helping statements for the Round 2 survey if they were ideas that had not been included in the Round 1 survey and were within the scope of the project. The Round 2 survey also included items from Round 1 that needed re-rating. Prior to being sent the survey, participants were emailed a report with a summary of the ratings of each panel for items to be re-rated, along with a list of items that were endorsed and rejected. This allowed participants to compare their ratings against each panel’s rating and consider whether they wished to change or keep their answer the same in the next round [35].

Rounds 2 and 3 did not provide an opportunity for further comments. If a re-rated item was not rated as ‘essential’ or ‘important’ by 80 % or more of each panel, it was rejected. Statements that met criteria for a re-rate in Round 2 were included in the final Round 3 survey. Participants were emailed four reminders to complete the survey over a 4-week period in each Round.

### Guidelines formation

The statements that reached the consensus criterion were written into connected sentences and edited by the working group. The final draft was shown to participants to obtain feedback on style and clarity. Comments received were used to finalise the wording of the guidelines.

### Ethics

The Human Research Ethics Committee of the University of Melbourne approved the research in July 2015. Informed consent was obtained from all participants by clicking ‘yes’ to a question about informed consent in the Round 1 survey.

## Results

A total of 141 experts were recruited, with 89 completing all three rounds - 23 consumers, 26 managers and 38 workplace mental health professionals. The retention rate for completing all three rounds was 61.7 % (see Table 3 for a breakdown according to panel type). Participants who completed all three rounds were 79.3 % female, 19.6 % male and 1.1 % identified as transsexual female. They were on average 47.5 years of age (10.3 SD, range 23 – 70) and were from Australia (93.1 %), the United States (3.6 %), Canada (1.1 %), New Zealand (1.1 %) and the United Kingdom (1.1 %). Of the 23 consumers, there were 4 managers, 3 consumer consultants/advocates, 3 policy officers/advisors, 2 nurses, 2 students, 2 academics, 2 administrators, 1 teacher, 1 consultant, 1 patient services assistant, 1 self-employed person, 1 retired mental health professional, 1 person on a disability support pension and 2 who did not specify their employment status or occupation. Of the 38 workplace mental health professionals, there were 11 workplace health and safety professionals, 9 trainers, 4 psychologists, 4 social workers, 3 workplace mental health program managers, 2 researchers, 2 nurses, 1 Employee Assistance Program Co-ordinator, 1 mental health advisor, and 1 manager.

**Table 3:** Retention rate

Expert panel	Round 1	Round 2	Round 3	Retention rate
Consumers	43	27	23	53.4 %
Managers	35	28	26	74.3 %
Workplace Mental Health Professionals	63	43	38	60.3 %
Total	141	98	87	61.7 %

### Item ratings

**Fig. 1:**
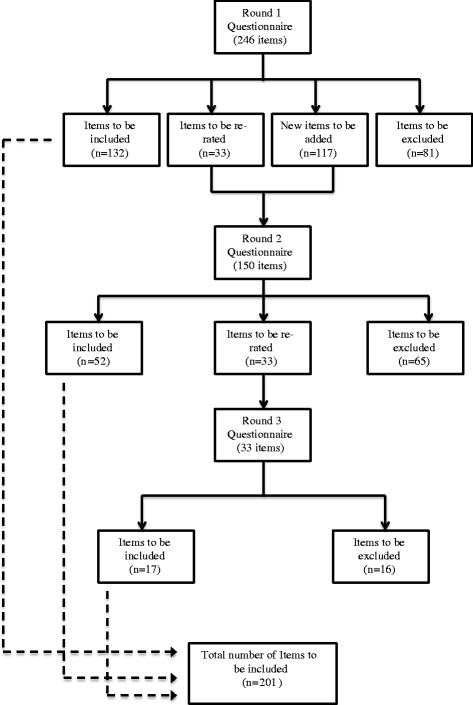
Total number of items endorsed, re-rated and rejected

A total of 363 items were rated over 3 rounds, which resulted in 201 endorsed items and 162 rejected items. Figure 1 illustrates the total number of items endorsed, re-rated and rejected over the three rounds (also see Additional file 2: for a list of the endorsed and rejected items). Overall, ratings of whether items should be included in the guidelines were similar across the consumer, manager and professional panels, with correlations for item endorsement rates of 0.88 between consumers and managers, 0.86 between consumers and workplace mental health professionals and 0.92 between managers and workplace mental health professionals.

The endorsed items outline what a co-worker and manager should do to help a co-worker showing signs of a mental health problem or experiencing a mental health crisis. They include knowing the signs and symptoms of a mental health problem at work and how work contributes to mental health problems. The guidelines outline specific actions for how to approach and talk with the person in a caring and non-judgmental way. They also provide guidance for how a person’s manager would offer mental health first aid, such as when reasonable adjustments or performance management are also required. Strategies for managing crisis situations, like acute distress and intoxication in the workplace, are also covered. See Table 4 for a summary of the number of items presented in each theme and the number of items endorsed.

**Table 4:** Themes and sub-themes of the guidelines

Themes	Sub-themes	Number of items	Number of endorsed items
General Awareness		6	6
Signs and symptoms of mental health problems in the workplace		4	3
Making the approach	Deciding whether to approach If the person’s behavior is affecting others or posing a risk to health and safety Planning your approach Additional considerations if you are the person’s manager	78	30
Listening and communicating non-judgmentally	Confidentiality Building trust When talking with the person Don’ts What if I’m not the right person to help What if they don’t want to talk? Additional considerations if you are the person’s manager	116	74
Giving support and information	Additional considerations if you are the person’s manager	100	49
Helping the distressed co-worker		22	18
Helping the intoxicated co-worker	Additional considerations if you are the person’s manager	28	13

As expected, there were some differences between groups. Items that were endorsed or rejected by one group only and received a notably higher or lower rating (±10 %) by this group are noted below. Though there is no set cut-off in Delphi studies, the cut-off is consistent with similar studies [28, 29, 35] and is greater than a small effect size [38].

### Comparison between consumers and other groups

There were two items that received lower ratings from consumers compared to managers and workplace mental health professionals. These were: ‘the manager should inform the person of the appropriate use of sick leave entitlements’ and ‘the helper should not organise a team meeting to confront the person, even if all other attempts to help the person who is misusing substances fail.’

There were six items that received higher ratings from consumers compared to managers and workplace mental health professionals that generally fit into the following themes: approaching someone in a more senior role; communicating non-judgementally, non-verbally and verbally; talking to others; and giving support and information.

### Comparison between managers and other groups

There were no items rated lower by managers compared to the other groups and there was only one item that received a higher rating from managers compared to consumers and workplace mental health professionals. This item was: ‘if after following up with the manager the concerns are still not addressed satisfactorily, the helper should raise their concerns with the health and safety manager or representative’.

### Comparison between workplace mental health professionals and other groups

There were six items that received lower ratings from workplace mental health professionals compared to consumers and managers that generally fit into the following themes: confidentiality; approaching the person; and giving support and information.

### Guidelines development

The endorsed items formed the basis of guidelines for providing mental health first aid to a co-worker and are available to download from www.mhfa.com.au. See Additional file 3: for a copy of the guidelines.

## Discussion

This study used the Delphi method to develop guidelines on how to offer mental health first aid to a co-worker. Overall, 201 actions were endorsed from a range of options. Table 4 presents the main themes and sub-themes of the guidelines. The endorsed actions were written into a guidelines document which organisations can freely access to help inform their training, policies and practice. The guidelines provide instruction on when and how to approach a co-worker who may be experiencing a mental health problem or crisis, listening and communicating non-judgmentally, providing support and information, and how to respond in crisis situations. There are additional strategies to consider if the first aider is also the person’s manager.

The aim of this study was to complement the content in the existing guidelines by developing guidelines on how to tailor mental health first aid when it is provided in a workplace setting. There are already guidelines about how to offer assistance to someone who may be experiencing a range of mental health problems and crises [18–25]. However, some duplication of content did occur, for example with items around choosing a time and place to approach a person or building trust and listening non-judgmentally [23]. A challenge previously noted with developing guidelines for workplaces is making them specific enough to be useful, while still being broad enough to be applicable across various industries and types of workplaces [28]. There are some items that refer to services such as an Employer Assistance Program and Human Resources. These items may not be applicable for smaller businesses or organisations [39].

Of the seven themes covered by the Delphi items, there were three themes that generated many differences of opinion and commentary from the panellists. Firstly, the panellists often commented on the need for a clear delineation between when a person is carrying out the first aider role versus any other roles they may carry out in the organisation, e.g. they are the person’s manager. In the feedback received, many participants indicated that the applicability of some of the items varied depending on the workplace hierarchical relationship between the first aider and the recipient of the first aid. These comments led to the working group developing a number of items for the Round 2 survey that specified that the first aider is the person’s manager, this removed ambiguity and led to many of the revised items being endorsed. For example, the following items were endorsed when reworded to be acted on by the person’s manager: ‘After raising their concerns with the person, the MANAGER should ask if the person would like to continue the conversation in the presence of a support person, e.g. an external advocate, co-worker’ and ‘the MANAGER should be aware of any legal obligations that the person has to disclose that they have a mental health problem at work, e.g. medical practitioners may be required to report medical conditions that impair their performance.’ Role delineation was also a theme in differences found between groups. Items that were rejected by the workplace mental health professionals panel, such as asking how long the person has been experiencing a mental health problem, may have been rejected because they were seen to be more appropriate to be carried out by a mental health professional or an Employee Assistance Program provider.

The second theme that raised a lot of commentary amongst participants was in relation to privacy, both in the between panel differences, such as those rejected by the workplace mental health professionals, and in the items rejected by all panels. Breaking confidentiality was only endorsed when the health and safety of the person or other co-workers is at risk. The panellists rejected items related to breaking confidentiality if the person no longer fulfils the inherent requirements of the role or is risking the reputation of the workplace due to their mental health problems. Also, only two items were endorsed out of a possible eleven items on the theme of documentation, with the use of documentation only deemed appropriate when a worker is posing a risk to the health and safety of others, or if the person assisting is the worker’s manager.

With these findings in mind, if the workplace culture is unsupportive of workers with a mental illness, it could be quite risky to share concerns or information about a worker to a third party within an organisation. For example, one participant provided specific comment on the importance of raising issues directly with the person only: *“Confidentiality is a big concern here. Most employees experiencing a mental health issue do not want [it] to be discussed with others. It would have to be quite serious for me to involve other colleagues etc.”* Participants commented that when a person is providing mental health first aid to a co-worker, their primary concern should be for the person rather than the organisation. Thus, is it understandable that many items regarding the boundaries of confidentiality and the needs for documentation were rejected, because of the risk of how the information may be used. As another participant commented:

*“It’s difficult to answer really because a lot relies upon the culture that exists in the workplace. The last thing we would want is for people to ‘use’ this information to their advantage, e.g. if there’s a high level of competition that exists in the workplace. The motives need to be pure as the goal should always be to encourage people to access the help and support they need. The less people involved in that process the better in my view to respect a person’s privacy. If seeking advice not revealing the person’s name etc. is of utmost importance.”*

Finally, a theme that panellists found difficulty in reaching any consensus on concerned items that would provide guidance on what to do if the first aider did not feel comfortable to approach a person in a more senior position. Out of a possible eight actions, the only one endorsed was to seek advice from the Employee Assistance Program on what to do, whereas seeking assistance from other resources such as a co-worker at the same level as the person, the person’s manager or Human Resources were rejected. This finding draws attention to the complexity risks and potential conflicts of interest involved in helping people at different levels in the workplace hierarchy.

The guidelines may be compared to other workplace guidelines, including psychological health and safety standards developed in Canada, and prevention and return-to-work guidelines developed in Australia [28, 29, 40]. The focus and scope of these other workplace guidelines are quite different, in that they focus more on actions that can be taken by the organisation rather than an individual worker, and across a variety of areas rather than on the narrow area of mental health first aid. The guidelines can also be compared to other mental health first aid guidelines, such as those developed for helping someone with depression [23]. The current guidelines are complementary in that they cover additional things a co-worker should consider in relation to their role relative to the person requiring mental health first aid, supports the person can access within the workplace, and how to address help-seeking barriers in the workplace.

The study had a number of strengths. Three panels were used with diverse sources of expertise, which is a desirable feature in Delphi studies [35]. The panel types, consumers, managers and workplace mental health professionals, were also similar to those used in other workplace mental health Delphi studies [28, 29]. Also panellists were recruited from several English-speaking countries, which increases the generalisability of findings. A limitation was the dropout rate of panel members from 141 in the first round to 98 in the second, particularly within the consumer panel, which may have been due to survey fatigue, as the first Round survey took approximately 1 h to complete. However, the final size of each panel conformed to that recommended for Delphi studies [36]. Although the study aimed to recruit panellists from a number of English-speaking developed countries, the majority of participants were from Australia. However, it is more important that participants in Delphi study have diverse expertise rather than be representative [35]. Finally, several participants in the first survey round questioned whether the first aider was in an informal or formal role within the workplace. Although information was provided at the beginning of the survey explaining that the first aider was *not* necessarily in a formal role such as a ‘Mental Health First Aid Officer’, some participants may have completed the survey with this role in mind.

There were three main advantages of using the Delphi method for this study: firstly it is an appropriate methodology to use when other research evidence is unavailable on a topic; secondly expert consensus is a way of tapping into practice-based evidence, drawing on both professional and lived-experience expertise; and thirdly it can help answer a question that is not feasibly answered via other methodologies such as randomised controlled trials [35]. The chief disadvantage of using the Delphi method is that it does not provide any evidence on the effects of applying the strategies in the guidelines.

## Conclusions

Workers may be more comfortable to disclose a mental health problem if they and their co-workers have the confidence and skills in how to provide mental health first aid. Using the consensus of people with lived experience of mental health problems whilst working, managers and workplace mental health professionals, clear strategies have been identified on how to offer mental health first aid to someone at work. These guidelines can now be used to help inform future Mental Health First Aid training programs, and influence organisational policies and procedures. These guidelines add to a growing set of best practice guidelines and other resources that are being developed [28, 29, 41]. These guidelines and resources support workplace mental health programs that address mental health problems at work regardless of cause, prevent the adverse impacts of work on mental health, and promote the positive aspects of work on mental wellbeing [42]. These guidelines will assist workers affected by mental health problems by enhancing early recognition, early help-seeking and consequently enhancing the provision of timely and appropriate support which may in turn limit the possible impact mental health problems may have on their working life. Though the guidelines are aimed at individual workers better supporting one another, if employers encourage the training of these skills across an entire workplace, there could be productivity benefits for the workplace as a whole.

## Abbreviation

MHFA, mental health first aid

## Additional files

Additional file 1: Copies of the Round 1, 2 and 3 surveys. (PDF 1272 kb) Additional file 2: Endorsed and rejected items including the survey round that they were endorsed or rejected. (XLSX 166 kb) Additional file 3: Final guidelines document. (PDF 1277 kb)
