# Supplementary material for: Providing mental health first aid in the workplace: a Delphi consensus study
Source: BMC Psychol. 2016 Aug 2;4:41. doi: 10.1186/s40359-016-0148-x (PMC4971664; doi:10.1186/s40359-016-0148-x)
Supplement: Additional file 1: — Copies of the Round 1, 2 and 3 surveys. (PDF 1272 kb) [file 40359_2016_148_MOESM1_ESM.pdf]

How to help employees and co-workers with mental health problems

Information about this survey

Purpose of the research

The aim of this current research project is to develop guidelines for how to provide mental health first aid to a co-worker. Once developed, these guidelines will provide guidance for employees on giving appropriate support to a co-worker who is developing a mental health problem or experiencing a mental health crisis.

The project is being conducted by Professor Tony Jorm, A/Prof Tony LaMontagne, A/Prof Angela Martin and Nataly Bovopoulos (PhD candidate) at the School of Population and Global Health, The University of Melbourne. This project has been approved by the University's Human Research Ethics Committee (HREC#: 1339508.1).

Why have I been selected?

You have been selected as a panel member for this study because you have expertise with the workplace and mental health (either as a manager, professional or consumer).

What do I need to do?

Your task is to rate the statements presented in this questionnaire according to how important you believe they are to providing mental health first aid to someone in the workplace. When rating the statements, please keep in mind that the guidelines will be used by any employee in a workplace and as such, the statements need to be rated according to how important each one is for someone, who does not necessarily have a counselling or clinical background, to provide mental health first aid to another employee. *Note: In each section, there are some items that are actions for managers only.*

For example you may be asked to rate the following statement:

1. The helper should not hurry the person because this can cause them anxiety.

You will need to decide how important, from *'essential'* to *'should not be included',* you think it is that this statement be included in the guidelines.

For an example of previously developed guidelines go to: First aid guidelines for assisting the person who is suicidal here.

How to help employees and co-workers with mental health problems

Information about this survey (cont)

What if something important is missing or if I have something to add?

There is a place for you to add comments at the end of each page. This is so that you can add any missing items or topics you think should be included in the guidelines. These items will then go into a second round of this survey to be rated by all of the expert panel members.

Where do the statements in the questionnaire come from?

The statements in the following questionnaire were derived from information found online, in books and in academic journal articles. Some of the statements may seem contradictory or controversial; however, we have included them because they reflect the wide range of people's beliefs. It is important to note that we do not necessarily agree with these statements; we have included them because we do not believe that we should decide what the best practice is in this area. Rather, we want an expert panel to decide this.

What is the focus of the questionnaire?

The questionnaire will focus on how an employee can provide mental health first aid to a fellow employee who may be developing a mental health problem or experiencing a mental health crisis.

How long will it take to complete the questionnaire?

This questionnaire should take approximately 60 minutes to complete. However, some people may finish it more quickly, while others may take longer, especially if they wish to lodge comments at the end of each section.

Can I save my answers and come back to the questionnaire?

If necessary, you can complete the survey in two or more sittings. You can save your answers at any time by completing a page and clicking 'Next' at the bottom. This marks your page as complete and you can begin again at a later date on the next page. Please make sure that you always log back in using the **same computer,** and that cookies are enabled, otherwise the software will fail to recognise your code and previously saved responses.

How to help employees and co-workers with mental health problems

Consent to participate in this research

If you have concerns about the project

If you have concerns about the scientific aspects of the study, please contact Professor Tony Jorm (ajorm@unimelb.edu.au, +61 3 9035 7799)

If you have concerns about the ethics of the study, please contact: Manager, Human Research Ethics, The University of Melbourne, ph: +61 3 8344 2073

Consent to participate

It is important for you to know that participation in this study is completely voluntary. You are not under any obligation to participate and you can withdraw at any time.

We would like to thank you for your time and effort and encourage you to provide us with feedback on this process.

Are you eligible to participate?

To participate in this research you must:

• **Have a lived experience of mental health problems whilst working** , your mental health symptoms are well managed at the moment, and you have experience in an advocacy role.

OR

• **Have a minimum of 5 years experience managing other employees** , with either training in mental health or practical experience supervising an employee with a mental health problem.

OR

• **Have a minimum 5 years experience specialising in workplace mental health or workplace health** in a clinical, policy or program role.

* 1. Please tick the appropriate answer.

- ◯ Yes, I meet this criteria.
- ◯ No, I do not meet this criteria.

How to help employees and co-workers with mental health problems

Consent to participate in this research (cont.)

* 2. I understand that by submitting this survey I am giving my consent to participate in this study.

- ◯ Yes, I understand.

How to help employees and co-workers with mental health problems

Information about you

* 3. Which best describes your area of expertise:

- ◯ I have lived experience of mental health problems whilst working, with my symptoms currently being well managed and experience in an advocacy role
- ◯ I am a manager with experience in the supervision of an employee with a mental health problem and/or have completed training in mental health
- ◯ I am a workplace mental health and/or workplace health professional

* 4. Do you live and, if applicable, work in one of the following countries:

- Australia
- Canada
- Ireland
- New Zealand
- The United Kingdom
- USA
- None of the above

How to help employees and co-workers with mental health problems

Information about you (cont)

* 5. Where do you live?

City/Town:

State/Province:

* 6. Are you at least 18 years old?

- Yes
- No

How to help employees and co-workers with mental health problems

Information about you (cont)

* 7. How old are you?

* 8. What is your gender?

- Female
- Male
- Gender queer
- Other (please specify)

9. If applicable, what is your occupation and title?

* 10. Please name all the relevant organisations you are affiliated with and your role within these organisations, e.g. professional organisations, peer support programs, advocacy groups.

How to help employees and co-workers with mental health problems

Overview of survey content

This survey is divided into the following sections:

1. General Awareness

2. Approach

3. Communication

4. Performance

5. Follow-up

6. Distress

7. Support

8. Reasonable adjustments

9. Disclosure

10. Substance use problems and intoxication

How to help employees and co-workers with mental health problems

Definitions of terms used in this survey

These terms may have different meanings for participants. The definitions below are how these terms are used in this survey. When completing the survey, please ensure you use these definitions only.

Common mental health problems in this survey refers to signs and symptoms of problematic anxiety, depression or substance use.

Person refers to an employee with signs and symptoms of a common mental health problem.

Mental health first aid means the help provided by any employee to another employee who is developing a mental health problem or experiencing a mental health crisis. The help is provided until professional help is received or until the crisis resolves. This help may be provided by one person or by a range of different people.

Helper refers to any employee who provides mental health first aid as defined above. This helper does not necessarily have a designated role, such as a physical first aid officer has. The helper may be a co-worker of the person, be the person's manager, or in a role less senior to the person.

Workplace refers to a place of employment.

Employee refers to a person working for another person, business or organisation (who could also be working as a contractor or volunteer).

Manager refers to a person responsible for the supervision of one or more employees.

Workplace stressors refer to working conditions that can contribute to mental health problems, e.g., long working hours, excessive workload, poor supervisory support.

Reasonable adjustments refers to changes to the work environment that allow a person with a mental illness to work safely and productively.

Disclosure refers to the degree of information being shared with others at work about a person's mental health problem, e.g. functional limitations through to diagnosis.

Employee Assistance Programs refer to a free and confidential counselling service offered by employers to their employees to support their wellbeing in the workplace and in their personal lives.

How to help employees and co-workers with mental health problems

Section 1: General Awareness

This section contains statements about general awareness of mental health problems in the workplace.

There are already guidelines to help people who may be experiencing a range of mental health problems (click here to access these guidelines). We do not wish to replicate existing MHFA guidelines. Rather, our aim is to develop guidelines on how to tailor mental health first aid for a person assisting a co-worker who is experiencing mental health problem in the workplace.

Please rate how important (from 'essential' to 'should not be included') you think it is that each statement be included in the guidelines.

Please keep the definitions in mind when answering the questions.

* 11. If the helper is aware that the person has a history of mental health problems, they should not assume that any poor performance is necessarily due to these problems.

Essential	Important	Don't know/Depends	Unimportant	Should not be included
◯	◯	◯	◯	◯

* 12. The helper should remember that not all workplace stressors affect everyone in the same way.

Essential	Important	Don't know/Depends	Unimportant	Should not be included
◯	◯	◯	◯	◯

* 13. The helper should be aware:

	Essential	Important	Don't know/ Depends	Unimportant	Should not be included
Of basic information about mental health problems e.g. what a mental health problem is, how common they are, the warning signs and symptoms, risk factors etc	○	○	○	○	○
Of the impact of the symptoms of mental health problems on the skills necessary for work, e.g. problems with concentration, memory, decision making and motivation	○	○	○	○	○
Of how stigma and discrimination can affect people with mental health problems in the workplace	○	○	○	○	○
Of the barriers to help-seeking for a person with a mental health problem	○	○	○	○	○
Of the pros and cons for employees disclosing any mental health problems at work	○	○	○	○	○
That most people with mental health problems who receive treatment respond with improved work performance	○	○	○	○	○
That while some people with mental health problems manage their symptoms without impact on their work performance, others may require short-term or ongoing workplace supports	○	○	○	○	○
That the level of support the person may need will fluctuate, as the symptoms of most mental health problems come and go over time	○	○	○	○	○
That investing time and support to retain an experienced and skilled employee with mental health problems is cheaper than recruiting and training a new person	○	○	○	○	○
Of factors other than mental health problems that contribute to problem behaviours in the workplace, e.g. poor performance, interpersonal conflict	○	○	○	○	○
Of the ways in which mental health problems may make a person a better employee, e.g. more aware of their own strengths and weaknesses, more empathic towards co-workers	○	○	○	○	○
That work can be bad or good for the person's mental health depending on the circumstances	○	○	○	○	○
Of the value of work for the person's recovery	○	○	○	○	○
Of the relevant laws and organisational policies that are relevant to employees with mental health problems.	○	○	○	○	○

14. Do you have any additional statements that you would like to add in this section? Please write your suggestions in the box provided.

How to help employees and co-workers with mental health problems

Section 2: Approach

This section contains statements about approaching the person to talk about their concerns.

There are already guidelines to help people who may be experiencing a range of mental health problems (click here to access these guidelines). We do not wish to replicate existing MHFA guidelines. Rather, our aim is to develop guidelines on how to tailor mental health first aid for a person assisting a co-worker who is experiencing mental health problem in the workplace.

Please rate how important (from 'essential' to 'should not be included') you think it is that each statement be included in the guidelines.

Please keep the definitions in mind when answering the questions.

* 15. The helper should look for the signs and symptoms at work that may indicate that the person is experiencing mental health problems.

Essential	Important	Don't know/Depends	Unimportant	Should not be included
○	○	○	○	○

* 16. The helper should not normalise problem behaviours as part of the person's personality, as this may make it difficult to recognise mental health problems.

Essential	Important	Don't know/Depends	Unimportant	Should not be included
○	○	○	○	○

* 17. If the person behaves inappropriately in the workplace, the helper should consider that the behaviour may be due to a mental health problem.

Essential	Important	Don't know/Depends	Unimportant	Should not be included
○	○	○	○	○

* 18. The helper should consider the following in deciding whether to approach the person:

	Essential	Important	Don't know/ Depends	Unimportant	Should not be included
Whether they have the appropriate knowledge and skills to assist the person	◯	◯	◯	◯	◯
Whether they have any negative experiences, attitudes or beliefs towards people with mental health problems	◯	◯	◯	◯	◯
If they are the person's manager, whether there is a conflict between their management and first aid roles	◯	◯	◯	◯	◯
The extent to which their working role is being affected by the person's behaviour	◯	◯	◯	◯	◯
The nature and quality of their working relationship	◯	◯	◯	◯	◯
Whether the workplace culture is contributing to the person's mental health problem.	◯	◯	◯	◯	◯

* 19. If after considering the above, the helper thinks that it is not appropriate for them to approach the person, they should consider talking about the situation with someone who may be in a better position to help and asking them to approach the person.

Essential	Important	Don't know/Depends	Unimportant	Should not be included
◯	◯	◯	◯	◯

* 20. If there is no one else available or appropriate to help, the helper should:

	Essential	Important	Don't know/ Depends	Unimportant	Should not be included
Approach the person regardless of whether they think they are the ideal person to help	◯	◯	◯	◯	◯
Talk about the situation with someone else who can advise on how to approach the person	◯	◯	◯	◯	◯
Talk with a mental health professional about how to approach the person.	◯	◯	◯	◯	◯

* 21. Even if the person is in a role that is senior to the helper, the helper should approach them or speak to another employee who can make the approach.

Essential	Important	Don't know/Depends	Unimportant	Should not be included
◯	◯	◯	◯	◯

* 22. If the person is in a role that is senior to the helper and they do not feel they can make the approach, they should consider:

	Essential	Important	Don't know/ Depends	Unimportant	Should not be included
Contact their professional organisation or union for advice	◯	◯	◯	◯	◯
Speak to the person's co-worker who is at the same level in the workplace, e.g. another middle manager	◯	◯	◯	◯	◯
Speak to the person's manager	◯	◯	◯	◯	◯
Speak to HR.	◯	◯	◯	◯	◯

23. Do you have any additional statements that you would like to add in this section? Please write your suggestions in the box provided.

How to help employees and co-workers with mental health problems

Section 2: Approach (cont)

This section contains statements about approaching the person to talk about their concerns.

There are already guidelines to help people who may be experiencing a range of mental health problems (click here to access these guidelines). We do not wish to replicate existing MHFA guidelines. Rather, our aim is to develop guidelines on how to tailor mental health first aid for a person assisting a co-worker who is experiencing mental health problem in the workplace.

Please rate how important (from ‘essential’ to ‘should not be included’) you think it is that each statement be included in the guidelines.

Please keep the definitions in mind when answering the questions.

* 24. The helper should approach the person at the first signs of distress or difficulty at work, because early intervention can make mental health problems easier to resolve.

Essential	Important	Don't know/Depends	Unimportant	Should not be included
◯	◯	◯	◯	◯

* 25. If the helper notices worrying changes in the person's work or interactions with others, they should discuss this with them.

Essential	Important	Don't know/Depends	Unimportant	Should not be included
◯	◯	◯	◯	◯

* 26. It is important for the helper to approach the person showing signs of a mental health problem, whether or not work is a contributor.

Essential	Important	Don't know/Depends	Unimportant	Should not be included
○	○	○	○	○

* 27. If the person is showing signs or symptoms of mental health problems, the helper should offer assistance, even if the person is still working productively.

Essential	Important	Don't know/Depends	Unimportant	Should not be included
○	○	○	○	○

* 28. The helper should approach the person even if they are fearful of the outcome, e.g. that the person will respond negatively, that it will create trouble for the person, or that it will affect their working relationship.

Essential	Important	Don't know/Depends	Unimportant	Should not be included
○	○	○	○	○

* 29. If the person's mental health problems are posing a risk to the health and safety of themselves or others in the workplace, the helper should:

	Essential	Important	Don't know/ Depends	Unimportant	Should not be included
Document their concerns, including time, date and location of concerning behaviours.	○	○	○	○	○
Talk to an appropriate manager about the concerning behaviours.	○	○	○	○	○

* 30. The helper should discuss their concerns with other employees who may be affected by the person's behaviour, such as other team members or managers.

Essential	Important	Don't know/Depends	Unimportant	Should not be included
○	○	○	○	○

* 31. If the helper is uncertain whether the person has mental health problems, the helper should ask other coworkers if they have observed any concerning behaviours.

Essential	Important	Don't know/Depends	Unimportant	Should not be included
○	○	○	○	○

* 32. The helper should **approach a supervisor** if the person's behaviour is affecting others in the workplace, focusing on the behaviour rather than the causes.

Essential	Important	Don't know/Depends	Unimportant	Should not be included
○	○	○	○	○

* 33. The helper should **approach the person** if their behaviour is affecting others in the workplace, focusing on the behaviour rather than the causes.

Essential	Important	Don't know/Depends	Unimportant	Should not be included
○	○	○	○	○

* 34. If the helper has discussed their concerns with the manager, the helper should follow up with the manager to be sure the concerns were addressed.

Essential	Important	Don't know/Depends	Unimportant	Should not be included
○	○	○	○	○

* 35. If after following up with the manager, the concerns are still not addressed satisfactorily, the helper should raise their concerns with the manager's superior or the appropriate professional body or union.

Essential	Important	Don't know/Depends	Unimportant	Should not be included
○	○	○	○	○

36. Do you have any additional statements that you would like to add in this section? Please write your suggestions in the box provided.

How to help employees and co-workers with mental health problems

Section 2: Approach (cont)

This section contains statements about approaching the person to talk about their concerns.

There are already guidelines to help people who may be experiencing a range of mental health problems (click here to access these guidelines). We do not wish to replicate existing MHFA guidelines. Rather, our aim is to develop guidelines on how to tailor mental health first aid for a person assisting a co-worker who is experiencing mental health problem in the workplace.

Please rate how important (from ‘essential’ to ‘should not be included’) you think it is that each statement be included in the guidelines.

Please keep the definitions in mind when answering the questions.

Before approaching the person

* 37. Before the helper approaches the person, they should consider, what is the problem exactly and who is being directly affected.

Essential	Important	Don't know/Depends	Unimportant	Should not be included
○	○	○	○	○

* 38. Before approaching the person, the helper should:

	Essential	Important	Don't know/ Depends	Unimportant	Should not be included
Document any concerning behaviours that they have observed	○	○	○	○	○
Have on hand any documentation relating to work performance or observed behaviours	○	○	○	○	○
Consider the expected outcomes and plan the structure and aims of the discussion in advance	○	○	○	○	○
Make a plan for what they want to say	○	○	○	○	○
Think about a time and place to meet that best suits the helper and the person	○	○	○	○	○
Find out what resources are available in the workplace to support people with mental health problems, e.g., an Employee Assistance Program	○	○	○	○	○
Consider whether the problems they have observed are due to other factors, e.g. workplace culture or Interpersonal dynamics rather than mental health problems	○	○	○	○	○
Be familiar with the organisation's policies and procedures for accommodating employees with mental health problems.	○	○	○	○	○

* 39. The helper should use regular work activities as opportunities to talk with the person about their concerns, rather than holding a specific meeting focused on the person's mental health.

Essential	Important	Don't know/Depends	Unimportant	Should not be included
○	○	○	○	○

* 40. The helper should have the conversation with the person in a neutral private space, such as outside the office in a cafe, or somewhere where the person feels comfortable and conversations can't be overhead.

Essential	Important	Don't know/Depends	Unimportant	Should not be included
○	○	○	○	○

* 41. The helper should ensure the meeting place is free of distractions and interruptions, e.g. switch off mobile phones.

Essential	Important	Don't know/Depends	Unimportant	Should not be included
○	○	○	○	○

* 42. The helper should be prepared for the possibility that the person may not accept the support being offered.

Essential	Important	Don't know/Depends	Unimportant	Should not be included
○	○	○	○	○

43. Do you have any additional statements that you would like to add in this section? Please write your suggestions in the box provided.

How to help employees and co-workers with mental health problems

Section 2: Approach (cont)

There are already guidelines to help people who may be experiencing a range of mental health problems (click here to access these guidelines). We do not wish to replicate existing MHFA guidelines. Rather, our aim is to develop guidelines on how to tailor mental health first aid for a person assisting a co-worker who is experiencing mental health problem in the workplace.

Please rate how important (from 'essential' to 'should not be included') you think it is that each statement be included in the guidelines.

Please keep the definitions in mind when answering the questions.

The following items relate to how the person's MANAGER would approach the person.

* 44. Before approaching the person, the MANAGER should check any relevant organisational policies and procedures regarding performance problems if the person does not disclose a mental health problem.

Essential	Important	Don't know/Depends	Unimportant	Should not be included
○	○	○	○	○

* 45. If the organisation has no policies for accommodating employees with mental health problems, the MANAGER should consider the best way to accommodate the person in their role.

Essential	Important	Don't know/Depends	Unimportant	Should not be included
○	○	○	○	○

* 46. The MANAGER should approach the person directly and privately about their concerns, rather than indirectly with a group of employees, e.g. holding a meeting to talk about general concerns about 'some employees' or running training.

Essential	Important	Don't know/Depends	Unimportant	Should not be included
○	○	○	○	○

* 47. When approaching the person, the MANAGER should consider going to the person's worksite if they are a remote worker.

Essential	Important	Don't know/Depends	Unimportant	Should not be included
○	○	○	○	○

48. Do you have any additional statements that you would like to add in this section? Please write your suggestions in the box provided.

How to help employees and co-workers with mental health problems

Section 3: Communication

This section contains statements about communicating with the person in the workplace.

There are already guidelines to help people who may be experiencing a range of mental health problems (click here to access these guidelines). We do not wish to replicate existing MHFA guidelines. Rather, our aim is to develop guidelines on how to tailor mental health first aid for a person assisting a co-worker who is experiencing mental health problem in the workplace.

Please rate how important (from 'essential' to 'should not be included') you think it is that each statement be included in the guidelines.

Please keep the definitions in mind when answering the questions.

* 49. When talking with the person, the helper should:

	Essential	Important	Don't know/ Depends	Unimportant	Should not be included
Describe the specific observations and reasons for their concern	○	○	○	○	○
Avoid blame or accusation	○	○	○	○	○
Be familiar with the guidelines on how to help someone who is suicidal	○	○	○	○	○
Listen non-judgmentally	○	○	○	○	○
Demonstrate empathy and respect	○	○	○	○	○
Allow the person time to talk	○	○	○	○	○
Ask them what support they need right now	○	○	○	○	○
Offer information about available support	○	○	○	○	○
Allow sufficient time for the discussion with the person	○	○	○	○	○
Be aware that its more important to be motivated by care and concern than to say the right things	○	○	○	○	○
Be aware that they don't need to have all the answers	○	○	○	○	○
Say what feels comfortable for them	○	○	○	○	○
Stop and try again if what they say doesn't sound quite right, as it doesn't have to be the end of the conversation.	○	○	○	○	○

* 50. The helper should create a supportive and trusting environment when discussing mental health problems with the person by:

	Essential	Important	Don't know/ Depends	Unimportant	Should not be included
Sitting opposite the person	○	○	○	○	○
Sitting opposite the person, *but at a slight angle to the person*	○	○	○	○	○
Sitting an appropriate distance away, taking into account personal space	○	○	○	○	○
Sitting without any barriers between the helper and person, e.g. a desk	○	○	○	○	○
Sitting in a relaxed manner, without crossing arms *and legs*	○	○	○	○	○
Sitting in a relaxed manner, without crossing arms	○	○	○	○	○
Bending slightly forward towards the person	○	○	○	○	○
Engaging with the person by using culturally appropriate eye contact.	○	○	○	○	○

51. Do you have any additional statements that you would like to add in this section? Please write your suggestions in the box provided.

How to help employees and co-workers with mental health problems

Section 3: Communication (cont)

This section contains statements about communicating with the person in the workplace.

There are already guidelines to help people who may be experiencing a range of mental health problems (click here to access these guidelines). We do not wish to replicate existing MHFA guidelines. Rather, our aim is to develop guidelines on how to tailor mental health first aid for a person assisting a co-worker who is experiencing mental health problem in the workplace.

Please rate how important (from 'essential' to 'should not be included') you think it is that each statement be included in the guidelines.

Please keep the definitions in mind when answering the questions.

What to discuss

* 52. The helper should consider starting the conversation with the person by talking about how their own personal struggles have affected their work behaviour.

Essential	Important	Don't know/Depends	Unimportant	Should not be included
○	○	○	○	○

* 53. When talking with the person about their concerns, the helper should

	Essential	Important	Don't know/ Depends	Unimportant	Should not be included
First talk about the person's strengths and how they are valued	◯	◯	◯	◯	◯
Express their concerns in a non-confrontational and clear manner, using examples of what the helper feels are the concerning changes	◯	◯	◯	◯	◯
State changes they have observed in a positive way, e.g. "You are not your usual cheery self" or "you seem less energetic"	◯	◯	◯	◯	◯
Stay focused on work-related issues	◯	◯	◯	◯	◯
Focus the discussion on the person's health and wellbeing, rather than their work performance	◯	◯	◯	◯	◯
Ask questions in an open and exploratory way, e.g. "I've noticed that you've been arriving late recently and wondered if there was a problem?"	◯	◯	◯	◯	◯
Ask open-ended questions about how any mental health problems may be affecting the person	◯	◯	◯	◯	◯
Listen if the person is willing to discuss their mental health problems	◯	◯	◯	◯	◯
Be honest and open when the person's performance is being affected, even if they are worried that this may distress the person.	◯	◯	◯	◯	◯

* 54. After raising their concerns with the person, the helper should ask:

	Essential	Important	Don't know/ Depends	Unimportant	Should not be included
Whether the person would feel more comfortable discussing the problem with someone else	◯	◯	◯	◯	◯
If the person would like to continue the conversation in the presence of a support person, e.g. an external advocate, co-worker.	◯	◯	◯	◯	◯

* 55. The helper should ask the person

	Essential	Important	Don't know/ Depends	Unimportant	Should not be included
What impact any mental health problems are having on them	○	○	○	○	○
How long they been experiencing a mental health problem, to determine whether this is an ongoing issue or Something where an immediate action could assist the situation	○	○	○	○	○
Whether the person has ever been given a diagnosis of a mental illness	○	○	○	○	○
If they have an advance directive or management plan in the workplace in case of an episode, if the person discloses a mental illness	○	○	○	○	○
If there are any non-work related issues they may like to talk about or make the helper aware of	○	○	○	○	○
Whether any workplace or other issues are contributing to their mental health problems	○	○	○	○	○
Whether any workplace stressors are contributing to any mental health problem the person is experiencing.	○	○	○	○	○

* 56. If the person says that workplace stressors are contributing to their mental health problem, the helper should take the person's concern seriously.

Essential	Important	Don't know/Depends	Unimportant	Should not be included
○	○	○	○	○

* 57. The helper should respect the person's wishes if they do not wish to discuss non-work related issues.

Essential	Important	Don't know/Depends	Unimportant	Should not be included
○	○	○	○	○

* 58. If the person has opened up and shared their story, the helper should express their appreciation.

Essential	Important	Don't know/Depends	Unimportant	Should not be included
○	○	○	○	○

59. Do you have any additional statements that you would like to add in this section? Please write your suggestions in the box provided.

How to help employees and co-workers with mental health problems

Section 3: Communication (cont)

This section contains statements about communicating with the person in the workplace.

There are already guidelines to help people who may be experiencing a range of mental health problems (click here to access these guidelines). We do not wish to replicate existing MHFA guidelines. Rather, our aim is to develop guidelines on how to tailor mental health first aid for a person assisting a co-worker who is experiencing mental health problem in the workplace.

Please rate how important (from 'essential' to 'should not be included') you think it is that each statement be included in the guidelines.

Please keep the definitions in mind when answering the questions.

Things to avoid

* 60. The helper should not:

	Essential	Important	Don't know/ Depends	Unimportant	Should not be included
Diagnose the person with a mental illness					
Offer the person remedies or treatments					
Counsel the person					
Make the person talk about their mental health problems if they don't want to					
Ask questions that create pressure like 'What's wrong with you' or 'Are you stressed or something"					
Use diagnostic terminology unless the person uses it					
Rush in with another question without listening to the answer					
Tell the person what to do					
Be patronising					
Treat the person as an invalid					
Assume the person's problems will just go away					
Tell the person that they need to stay busy or get out more					
Tell the person to 'snap out of it' or to 'get your act together'					
Adopt an overly friendly attitude					
Say "I've had the same experience" unless they have					
Minimise the person's problems by comparing them to the helper's own problems or those of other people					
Minimise the problems the person may encounter as a result of their mental health problems					
Label the person's mental health problems as 'bad' behaviour, e.g. referring to a person who is frequently late as lazy.					

* 61. The helper should avoid

	Essential	Important	Don't know/ Depends	Unimportant	Should not be included
Embarrassing the person by saying things like 'everyone is noticing…'					
Making assumptions about whether any sickness absence of the person is 'genuine.'					

* 62. The helper should use non-stigmatising language when talking about the person, e.g. 'people with schizophrenia' rather than 'schizophrenics', 'people with substance use problems' rather than 'addicts.'

Essential	Important	Don't know/Depends	Unimportant	Should not be included
◯	◯	◯	◯	◯

* 63. If the helper is unsure about what terminology to use when talking with the person about their mental health problem, they should ask the person what terms they use themselves.

Essential	Important	Don't know/Depends	Unimportant	Should not be included
◯	◯	◯	◯	◯

64. Do you have any additional statements that you would like to add in this section? Please write your suggestions in the box provided.

How to help employees and co-workers with mental health problems

Section 3: Communication (cont)

This section contains statements about communicating with the person in the workplace.

There are already guidelines to help people who may be experiencing a range of mental health problems (click here to access these guidelines). We do not wish to replicate existing MHFA guidelines. Rather, our aim is to develop guidelines on how to tailor mental health first aid for a person assisting a co-worker who is experiencing mental health problem in the workplace.

Please rate how important (from ‘essential’ to ‘should not be included’) you think it is that each statement be included in the guidelines.

Please keep the definitions in mind when answering the questions.

Confidentiality

* 65. The helper should be clear with the person about the limits of confidentiality.

Essential	Important	Don't know/Depends	Unimportant	Should not be included
◯	◯	◯	◯	◯

* 66. The helper should discuss and agree with the person about any limits on confidentiality and who will be told what, such as the helper needing to seek advice from OH&S, HR or others.

Essential	Important	Don't know/Depends	Unimportant	Should not be included
◯	◯	◯	◯	◯

* 67. The helper should maintain the person's confidentiality and privacy except where there is a concern:

	Essential	Important	Don't know/ Depends	Unimportant	Should not be included
For the safety of the person or others	○	○	○	○	○
That the person cannot fulfil their inherent requirements of the role	○	○	○	○	○
For harm to the organisation, e.g. reputational harm.	○	○	○	○	○

68. Do you have any additional statements that you would like to add in this section? Please write your suggestions in the box provided.

How to help employees and co-workers with mental health problems

Section 3: Communication (cont)

This section contains statements about communicating with the person in the workplace.

There are already guidelines to help people who may be experiencing a range of mental health problems (click here to access these guidelines). We do not wish to replicate existing MHFA guidelines. Rather, our aim is to develop guidelines on how to tailor mental health first aid for a person assisting a co-worker who is experiencing mental health problem in the workplace.

Please rate how important (from 'essential' to 'should not be included') you think it is that each statement be included in the guidelines.

Please keep the definitions in mind when answering the questions.

If they don't want to talk

* 69. The helper should be aware that the person may be reluctant to talk about any mental health problems for various reasons, e.g. fears about losing their job or other co-workers finding out, it doesn't significantly affect their work or because they have found ways to manage things on their own.

Essential	Important	Don't know/Depends	Unimportant	Should not be included
○	○	○	○	○

* 70. If the person is reluctant to talk, the helper should discuss the reasons for this reluctance and ways to deal with them, if appropriate.

Essential	Important	Don't know/Depends	Unimportant	Should not be included
○	○	○	○	○

* 71. If the person chooses not to talk, the helper should:

	Essential	Important	Don't know / Depends	Unimportant	Should not be included
Tell them that they are available to talk in the future if the person wishes to	○	○	○	○	○
Encourage them to talk to someone else they trust instead	○	○	○	○	○
Not take it personally.	○	○	○	○	○

* 72. The helper should remain calm if the person reacts in a negative way, e.g. denial, anger.

Essential	Important	Don't know/Depends	Unimportant	Should not be included
○	○	○	○	○

73. Do you have any additional statements that you would like to add in this section? Please write your suggestions in the box provided.

How to help employees and co-workers with mental health problems

Section 3: Communication (cont)

This section contains statements about communicating with the person in the workplace.

There are already guidelines to help people who may be experiencing a range of mental health problems (click here to access these guidelines). We do not wish to replicate existing MHFA guidelines. Rather, our aim is to develop guidelines on how to tailor mental health first aid for a person assisting a co-worker who is experiencing mental health problem in the workplace.

Please rate how important (from ‘essential’ to ‘should not be included’) you think it is that each statement be included in the guidelines.

Please keep the definitions in mind when answering the questions.

Communicating with other workers

* 74. The helper should not complain to other employees or spread rumours about the person behind their back.

Essential	Important	Don't know/Depends	Unimportant	Should not be included
○	○	○	○	○

* 75. When the helper is talking with another employee about their concerns about the person, they should be specific about their observations and avoid critical comments, e.g. "I have seen Lin engaged in heated arguments with two co-workers and even a client in the past week" rather than "Lin has gone crazy."

Essential	Important	Don't know/Depends	Unimportant	Should not be included
○	○	○	○	○

* 76. When the helper is talking with another employee who is concerned about the person, the helper should focus on possible solutions or support rather than solely on the problem.

Essential	Important	Don't know/Depends	Unimportant	Should not be included
○	○	○	○	○

* 77. If the helper hears other employees talking about the details of the person's mental health problems in an unhelpful way, they should let them know it is inappropriate.

Essential	Important	Don't know/Depends	Unimportant	Should not be included
○	○	○	○	○

* 78. If the person takes sick days and other co-workers feel that this is unfair, the helper should ask them if they would feel differently if the person were taking sick days for a physical injury/illness.

Essential	Important	Don't know/Depends	Unimportant	Should not be included
○	○	○	○	○

79. Do you have any additional statements that you would like to add in this section? Please write your suggestions in the box provided.

How to help employees and co-workers with mental health problems

Section 4: Performance

There are already guidelines to help people who may be experiencing a range of mental health problems (click here to access these guidelines). We do not wish to replicate existing MHFA guidelines. Rather, our aim is to develop guidelines on how to tailor mental health first aid for a person assisting a co-worker who is experiencing mental health problem in the workplace.

Please rate how important (from ‘essential’ to ‘should not be included’) you think it is that each statement be included in the guidelines.

Please keep the definitions *in mind when answering the questions.*

The following items relate tot how the person’s MANAGER would approach performance management and mental health first aid.

* 80. The MANAGER should be aware that behaviours that breach the workplace's rules or regulations may require both performance management and mental health first aid.

Essential	Important	Don't know/Depends	Unimportant	Should not be included
◯	◯	◯	◯	◯

* 81. If there are any performance or behaviour issues, the MANAGER should explain clearly what is expected of the person in terms of work performance and behaviour.

Essential	Important	Don't know/Depends	Unimportant	Should not be included
◯	◯	◯	◯	◯

* 82. If at any point it becomes apparent that the person's behaviours are breaching the workplace's rules or regulations, the MANAGER should suspend the meeting and explain that a formal interview will need to be arranged.

Essential	Important	Don't know/Depends	Unimportant	Should not be included
◯	◯	◯	◯	◯

* 83. If at any point it becomes apparent that the person's behaviours are breaching the workplace's rules or regulations and loss of job is imminent, the MANAGER should be clear about this and document the meeting as a performance issue so there is no confusion.

Essential	Important	Don't know/Depends	Unimportant	Should not be included
◯	◯	◯	◯	◯

84. Do you have any additional statements that you would like to add in this section? Please write your suggestions in the box provided.

How to help employees and co-workers with mental health problems

Section 5: Follow-up

This section contains statements about following-up the person in the workplace after making the

approach.

There are already guidelines to help people who may be experiencing a range of mental health problems (click here to access these guidelines). We do not wish to replicate existing MHFA guidelines. Rather, our aim is to develop guidelines on how to tailor mental health first aid for a person assisting a co-worker who is experiencing mental health problem in the workplace.

Please rate how important (from 'essential' to 'should not be included') you think it is that each statement be included in the guidelines.

Please keep the definitions in mind when answering the questions.

* 85. At the end of the conversation, the helper should agree with the person what will happen next and who will take action.

Essential	Important	Don't know/Depends	Unimportant	Should not be included
○	○	○	○	○

* 86. The helper should:

	Essential	Important	Don't know / Depends	Unimportant	Should not be included
Keep a record of all conversations about the person's mental health problems	○	○	○	○	○
Not keep a record of any conversation about the person's mental health problems without their permission	○	○	○	○	○
Give a copy of the conversation record to the person and check its accuracy with them	○	○	○	○	○
Keep a record of any actions taken as a result of conversations with the person	○	○	○	○	○
Make arrangements for a follow up meeting to review the situation	○	○	○	○	○
Informally check in with the person to see how they are doing.	○	○	○	○	○

* 87. If the helper is unable to follow up with the person, they should consider asking someone else to do this on their behalf, after seeking permission from the person.

Essential	Important	Don't know/Depends	Unimportant	Should not be included
○	○	○	○	○

* 88. If after the conversation the helper feels distressed, they should find someone to talk to for support and advice, while respecting the person's privacy.

Essential	Important	Don't know/Depends	Unimportant	Should not be included
○	○	○	○	○

* 89. If the helper's expectations of the discussion are not met, they should:

	Essential	Important	Don't know/ Depends	Unimportant	Should not be included
Try to speak to the person again at another time	○	○	○	○	○
Be aware that their actions may still make a difference, e.g. the person may speak to someone else about their mental health problem.	○	○	○	○	○

90. Do you have any additional statements that you would like to add in this section? Please write your suggestions in the box provided.

How to help employees and co-workers with mental health problems

Section 6: Distress

This section contains statements about supporting the person who is distressed in the workplace.

There are already guidelines to help people who may be experiencing a range of mental health problems (click here to access these guidelines). We do not wish to replicate existing MHFA guidelines. Rather, our aim is to develop guidelines on how to tailor mental health first aid for a person assisting a co-worker who is experiencing mental health problem in the workplace.

Please rate how important (from ‘essential’ to ‘should not be included’) you think it is that each statement be included in the guidelines.

Please keep the definitions in mind when answering the questions.

* 91. If the person is distressed, the helper should not ignore the person, as doing nothing can make the situation worse.

Essential	Important	Don't know/Depends	Unimportant	Should not be included
○	○	○	○	○

* 92. If the person is distressed, the helper should do the following (where appropriate to the circumstances and the helper's work role):

	Essential	Important	Don't know/ Depends	Unimportant	Should not be included
Let the person know they are listening	○	○	○	○	○
Tell them that help and support will be offered	○	○	○	○	○
Be aware that the person may not be able to think clearly and take in much information	○	○	○	○	○
Focus on what is distressing the person right now	○	○	○	○	○
Ask the person if they need them to contact someone on their behalf	○	○	○	○	○
Assess for any crisis (immediate risk of harm to themselves or others) and respond according to existing guidelines	○	○	○	○	○
Not feel they need to offer the person an immediate solution, especially if it has long-term implications	○	○	○	○	○
Not send the person home straight away	○	○	○	○	○
Reassure them that is it ok to be upset	○	○	○	○	○
Provide an appropriate space where they can express emotion freely and compose themselves in privacy	○	○	○	○	○
Suggest you both leave the workplace for a short time to have a coffee or short walk to give the person time to collect themselves	○	○	○	○	○
Let them know that once they have recovered sufficiently, they can carry on working, take a break, or seek permission to go home	○	○	○	○	○
Tell the person their job is safe	○	○	○	○	○
Reassure the person they are valued, as they may be feeling embarrassed or ashamed about what happened.	○	○	○	○	○

* 93. If needed, the helper should seek assistance to more effectively support a person who is distressed, while maintaining the person's privacy.

Essential	Important	Don't know/Depends	Unimportant	Should not be included
○	○	○	○	○

* 94. If needed, the helper should seek support**for themselves** after assisting a person who is distressed, while maintaining the person's confidentiality.

Essential	Important	Don't know/Depends	Unimportant	Should not be included
○	○	○	○	○

* 95. If the person is too distressed to talk, the helper should reassure the person that the discussion can continue at another time and place that suits them.

Essential	Important	Don't know/Depends	Unimportant	Should not be included
○	○	○	○	○

96. Do you have any additional statements that you would like to add in this section? Please write your suggestions in the box provided.

How to help employees and co-workers with mental health problems

Section 7: Support

This section contains statements about providing support to the person.

There are already guidelines to help people who may be experiencing a range of mental health problems (click here to access these guidelines). We do not wish to replicate existing MHFA guidelines. Rather, our aim is to develop guidelines on how to tailor mental health first aid for a person assisting a co-worker who is experiencing mental health problem in the workplace.

Please rate how important (from 'essential' to 'should not be included') you think it is that each statement be included in the guidelines.

Please keep the definitions in mind when answering the questions.

* 97. If the person discloses a mental illness, the helper should ask if they have an advance directive or management plan in the workplace in case of an episode.

Essential	Important	Don't know/Depends	Unimportant	Should not be included
○	○	○	○	○

* 98. If the person has an advance directive or management plan, the helper should follow the instructions.

Essential	Important	Don't know/Depends	Unimportant	Should not be included
○	○	○	○	○

* 99. The helper should encourage the person to use helpful coping strategies they have used in the past.

Essential	Important	Don't know/Depends	Unimportant	Should not be included
○	○	○	○	○

* 100. The helper should discuss how the workplace can support the person's existing coping strategies.

Essential	Important	Don't know/Depends	Unimportant	Should not be included
○	○	○	○	○

* 101. The helper should be aware that the person may not be able to think clearly about solutions.

Essential	Important	Don't know/Depends	Unimportant	Should not be included
○	○	○	○	○

* 102. If the person does not initially know what support they need, the helper should assist them in exploring the available options.

Essential	Important	Don't know/Depends	Unimportant	Should not be included
○	○	○	○	○

* 103. The helper should assist the person to identify workplace triggers that contribute to their mental health problems.

Essential	Important	Don't know/Depends	Unimportant	Should not be included
○	○	○	○	○

* 104. The helper should make contact with the person regularly in order to provide ongoing support and encouragement.

Essential	Important	Don't know/Depends	Unimportant	Should not be included
○	○	○	○	○

* 105. If the person is able to meet the majority of their job requirements, the helper should encourage them to keep coming to work.

Essential	Important	Don't know/Depends	Unimportant	Should not be included
○	○	○	○	○

* 106. The helper should assist the person to consider the pros and cons of taking time off work.

Essential	Important	Don't know/Depends	Unimportant	Should not be included
○	○	○	○	○

* 107. The helper should be aware that being at work in a supportive workplace can assist in the person's recovery.

Essential	Important	Don't know/Depends	Unimportant	Should not be included
○	○	○	○	○

* 108. The helper should not offer help to the person outside of the usual work roles, e.g. driving the person to their doctor's appointment.

Essential	Important	Don't know/Depends	Unimportant	Should not be included
○	○	○	○	○

109. Do you have any additional statements that you would like to add in this section? Please write your suggestions in the box provided.

How to help employees and co-workers with mental health problems

Section 7: Support (cont)

This section contains statements about providing support to the person.

There are already guidelines to help people who may be experiencing a range of mental health problems (click here to access these guidelines). We do not wish to replicate existing MHFA guidelines. Rather, our aim is to develop guidelines on how to tailor mental health first aid for a person assisting a co-worker who is experiencing mental health problem in the workplace.

Please rate how important (from 'essential' to 'should not be included') you think it is that each statement be included in the guidelines.

Please keep the definitions in mind when answering the questions.

* 110. The helper should **encourage** the person to seek professional help.

Essential	Important	Don't know/Depends	Unimportant	Should not be included
○	○	○	○	○

* 111. The helper should **offer to assist** the person to seek professional help.

Essential	Important	Don't know/Depends	Unimportant	Should not be included
○	○	○	○	○

* 112. If the person is reluctant to seek professional help, the helper should discuss the reasons for the person's reluctance.

Essential	Important	Don't know/Depends	Unimportant	Should not be included
○	○	○	○	○

* 113. The helper should make sure the person is aware of any relevant supports that the organisation can provide, e.g. EAP.

Essential	Important	Don't know/Depends	Unimportant	Should not be included
○	○	○	○	○

* 114. The helper should offer to assist the person to find information and support services.

Essential	Important	Don't know/Depends	Unimportant	Should not be included
○	○	○	○	○

* 115. The helper should encourage the person to consider:

	Essential	Important	Don't know/ Depends	Unimportant	Should not be included
Making changes at work to reduce stress levels	○	○	○	○	○
Talking to their manager about their concerns	○	○	○	○	○
Listing work tasks in order of priority	○	○	○	○	○
Telling loved ones about work problems and asking for their support.	○	○	○	○	○

* 116. If work-related stress continues to affect the person's mental health problems, the helper should encourage the person to consider changing jobs.

Essential	Important	Don't know/Depends	Unimportant	Should not be included
○	○	○	○	○

* 117. The helper should recommend evidence-based self-help strategies that are effective for managing mental health problems.

Essential	Important	Don't know/Depends	Unimportant	Should not be included
○	○	○	○	○

* 118. The helper should encourage the person who is withdrawing to interact with others at work.

Essential	Important	Don't know/Depends	Unimportant	Should not be included
○	○	○	○	○

119. Do you have any additional statements that you would like to add in this section? Please write your suggestions in the box provided.

Section 7: Support (cont.)

This section contains statements about providing support to the person.

There are already guidelines to help people who may be experiencing a range of mental health problems (click here to access these guidelines). We do not wish to replicate existing MHFA guidelines. Rather, our aim is to develop guidelines on how to tailor mental health first aid for a person assisting a co-worker who is experiencing mental health problem in the workplace.

Please rate how important (from 'essential' to 'should not be included') you think it is that each statement be included in the guidelines.

Please keep the definitions in mind when answering the questions.

Note: The following items relate to how the person's MANAGER would provide support to the person.

* 120. The MANAGER should ask the person what support they need in order to fulfil the inherent requirements of their job.

Essential	Important	Don't know/Depends	Unimportant	Should not be included
○	○	○	○	○

* 121. The MANAGER should encourage co-workers to support the person, whilst still respecting the person's privacy.

Essential	Important	Don't know/Depends	Unimportant	Should not be included
○	○	○	○	○

* 122. The MANAGER should try to adapt the way they manage the person in order to reduce any work-related stress, e.g. some people may function better with more direction from their manager.

Essential	Important	Don't know/Depends	Unimportant	Should not be included
○	○	○	○	○

* 123. The MANAGER should tell the person that they will help them get the support they need to return to their usual work performance.

Essential	Important	Don't know/Depends	Unimportant	Should not be included
○	○	○	○	○

* 124. If poor workplace relationships are contributing to the person's mental health problems, the MANAGER should consider using mediation to help resolve issues.

Essential	Important	Don't know/Depends	Unimportant	Should not be included
○	○	○	○	○

* 125. The MANAGER should be aware of the possible impact of the person's mental health problem on other employees and also offer them support.

Essential	Important	Don't know/Depends	Unimportant	Should not be included
○	○	○	○	○

* 126. The MANAGER should encourage the appropriate use of sick leave entitlements.

Essential	Important	Don't know/Depends	Unimportant	Should not be included
○	○	○	○	○

* 127. The MANAGER should with the person's permission, request medical reports or advice from their treating health professional.

Essential	Important	Don't know/Depends	Unimportant	Should not be included
○	○	○	○	○

* 128. The MANAGER should focus on encouraging help-seeking before they address any performance issues.

Essential	Important	Don't know/Depends	Unimportant	Should not be included
○	○	○	○	○

129. Do you have any additional statements that you would like to add in this section? Please write your suggestions in the box provided.

How to help employees and co-workers with mental health problems

Section 8: Reasonable Adjustments

This section contains statements about reasonable adjustments and the person.

There are already guidelines to help people who may be experiencing a range of mental health problems (click here to access these guidelines). We do not wish to replicate existing MHFA guidelines. Rather, our aim is to develop guidelines on how to tailor mental health first aid for a person assisting a co-worker who is experiencing mental health problem in the workplace.

Please rate how important (from 'essential' to 'should not be included') you think it is that each statement be included in the guidelines.

Please keep the definitions in mind when answering the questions.

* 130. The helper should tell the person that they are entitled to reasonable adjustments.

Essential	Important	Don't know/Depends	Unimportant	Should not be included
○	○	○	○	○

131. Do you have any additional statements that you would like to add in this section? Please write your suggestions in the box provided.

How to help employees and co-workers with mental health problems

Section 8: Reasonable Adjustments (cont)

This section contains statements about reasonable adjustments and the person.

There are already guidelines to help people who may be experiencing a range of mental health problems (click here to access these guidelines). We do not wish to replicate existing MHFA guidelines. Rather, our aim is to develop guidelines on how to tailor mental health first aid for a person assisting a co-worker who is experiencing mental health problem in the workplace.

Please rate how important (from 'essential' to 'should not be included') you think it is that each statement be included in the guidelines.

Please keep the definitions in mind when answering the questions.

The following items relate to how the person's MANAGER would provide reasonable adjustments for the person.

* 132. The MANAGER should be aware of relevant guidelines that are available on how to carry out reasonable adjustments for the person.

Essential	Important	Don't know/Depends	Unimportant	Should not be included
○	○	○	○	○

* 133. The MANAGER should provide an opportunity for the person to talk to someone, for when they might be feeling distressed at work, e.g. telephone help-line.

Essential	Important	Don't know/Depends	Unimportant	Should not be included
○	○	○	○	○

* 134. The MANAGER should use the following strategies (depending on the circumstances):

	Essential	Important	Don't know/Depends	Unimportant	Should not be included
Temporarily relaxing performance expectations for the person	○	○	○	○	○
Deferring 'bad news' until the person is feeling better	○	○	○	○	○
Having regular discussions with the person to monitor how they are coping with their workload and workplace accommodations.	○	○	○	○	○

* 135. The MANAGER should focus on performance management only after an agreed period of reasonable adjustments and after encouraging the person to seek professional help.

Essential	Important	Don't know/Depends	Unimportant	Should not be included
○	○	○	○	○

* 136. The MANAGER should explain to co-workers in a positive and supportive manner why any adjustments are being made.

Essential	Important	Don't know/Depends	Unimportant	Should not be included
○	○	○	○	○

* 137. The MANAGER should tell the person that they will not tolerate any harassment of the person due to their mental health problems.

Essential	Important	Don't know/Depends	Unimportant	Should not be included
○	○	○	○	○

138. Do you have any additional statements that you would like to add in this section? Please write your suggestions in the box provided.

How to help employees and co-workers with mental health problems

Section 9: Disclosure

This section contains statements about the person disclosing their mental health problem in the workplace.

There are already guidelines to help people who may be experiencing a range of mental health problems (click here to access these guidelines). We do not wish to replicate existing MHFA guidelines. Rather, our aim is to develop guidelines on how to tailor mental health first aid for a person assisting a co-worker who is experiencing mental health problem in the workplace.

Please rate how important (from 'essential' to 'should not be included') you think it is that each statement be included in the guidelines.

Please keep the definitions in mind when answering the questions.

* 139. The helper should be aware of any legal obligations that the person has to disclose that they have a mental health problem at work e.g. medical practitioners may be required to report medical conditions that impair their performance.

Essential	Important	Don't know/Depends	Unimportant	Should not be included
○	○	○	○	○

* 140. The helper should be aware of any relevant discrimination legislation in relation to the disclosure of mental health problems at work.

Essential	Important	Don't know/Depends	Unimportant	Should not be included
○	○	○	○	○

* 141. The helper should be aware of the possible barriers to disclosure, e.g. fear of discrimination, or fear of how their manager or co-workers may react.

Essential	Important	Don't know/Depends	Unimportant	Should not be included
○	○	○	○	○

* 142. The helper should be aware of the possible benefits of disclosure, e.g. reasonable adjustments, education of others, openness with others.

Essential	Important	Don't know/Depends	Unimportant	Should not be included
○	○	○	○	○

* 143. The helper should help the person to consider the pros and cons of disclosing mental health problems to co-workers and to managers.

Essential	Important	Don't know/Depends	Unimportant	Should not be included
○	○	○	○	○

144. Do you have any additional statements that you would like to add in this section? Please write your suggestions in the box provided.

How to help employees and co-workers with mental health problems

Section 9: Disclosure (cont)

This section contains statements about the person disclosing their mental health problem in the workplace.

There are already guidelines to help people who may be experiencing a range of mental health problems (click here to access these guidelines). We do not wish to replicate existing MHFA guidelines. Rather, our aim is to develop guidelines on how to tailor mental health first aid for a person assisting a co-worker who is experiencing mental health problem in the workplace.

Please rate how important (from ‘essential’ to ‘should not be included’) you think it is that each statement be included in the guidelines.

Please keep the definitions in mind when answering the questions.

Note: The following items relate to how the person’s MANAGER would approach disclosure issues.

* 145. The MANAGER should be aware of the legal, ethical and practical issues related to a person disclosing mental health problems.

Essential	Important	Don't know/Depends	Unimportant	Should not be included
○	○	○	○	○

* 146. The MANAGER should discuss with the person whether there is any information that co-workers need to be given about the person's mental health problems.

Essential	Important	Don't know/Depends	Unimportant	Should not be included
○	○	○	○	○

* 147. The MANAGER should discuss with the person what information they would like their co-workers to be given about the person's mental health problems.

Essential	Important	Don't know/Depends	Unimportant	Should not be included
○	○	○	○	○

* 148. Should the person not wish to disclose their mental health problems to co-workers, the MANAGER should:

	Essential	Important	Don't know/ Depends	Unimportant	Should not be included
Discuss with the person what adjustments are possible without making staff aware that the person has mental health problems	○	○	○	○	○
Try to prevent co-worker perception of 'special treatment' by disseminating policies on employee adjustments.	○	○	○	○	○

149. Do you have any additional statements that you would like to add in this section? Please write your suggestions in the box provided.

How to help employees and co-workers with mental health problems

Section 10: Substance use problems and intoxication

This section is about helping the person who has a substance use problem or is intoxicated in the workplace.

There are already guidelines to help people who may be experiencing a range of mental health problems (click here to access these guidelines). We do not wish to replicate existing MHFA guidelines. Rather, our aim is to develop guidelines on how to tailor mental health first aid for a person assisting a co-worker who is experiencing mental health problem in the workplace.

Please rate how important (from 'essential' to 'should not be included') you think it is that each statement be included in the guidelines.

Please keep the definitions in mind when answering the questions.

* 150. If the person uses substances outside of work hours, the helper should only approach the person about this if it impacts on the person's work performance.

Essential	Important	Don't know/Depends	Unimportant	Should not be included
○	○	○	○	○

* 151. If the person uses substances outside of work hours and this does not affect the person's work performance, the helper should still approach the person to let them know that they are concerned about their health.

Essential	Important	Don't know/Depends	Unimportant	Should not be included
○	○	○	○	○

* 152. If the person's substance use is putting co-workers at risk of harm, the helper should report this to a manager.

Essential	Important	Don't know/Depends	Unimportant	Should not be included
○	○	○	○	○

* 153. If all other attempts to help the person who is misusing substances fail, the helper should consider organising a team meeting to confront the person.

Essential	Important	Don't know/Depends	Unimportant	Should not be included
○	○	○	○	○

* 154. If the team is considering confronting the person, the helper should consider engaging a trained specialist to assist with this process.

Essential	Important	Don't know/Depends	Unimportant	Should not be included
○	○	○	○	○

* 155. If the person appears to be affected by drugs or alcohol while at work, the helper should:

	Essential	Important	Don't know/ Depends	Unimportant	Should not be included
Ensure that the person does not pose a health and safety risk to themselves or others	○	○	○	○	○
Escort the person to a private area	○	○	○	○	○
Call in a more senior co-worker to serve as a witness if possible	○	○	○	○	○
Inform the person of their concerns	○	○	○	○	○
Ask the person for any explanation for their behaviour, e.g. side-effects of a medication	○	○	○	○	○
Arrange for the person to be escorted home safely	○	○	○	○	○
Notify the police if the person insists on driving home.	○	○	○	○	○

* 156. The helper should not:

	Essential	Important	Don't know/Depends	Unimportant	Should not be included
Cover for the person who is affected by alcohol or other drugs e.g. carrying out the person's work tasks for them	○	○	○	○	○
Ignore the person's inappropriate substance use while at work.	○	○	○	○	○

157. Do you have any additional statements that you would like to add in this section? Please write your suggestions in the box provided.

How to help employees and co-workers with mental health problems

Section 10: Substance use problems and intoxication (cont)

This section is about helping the person who has a substance use problem or is intoxicated in the workplace.

There are already guidelines to help people who may be experiencing a range of mental health problems (click here to access these guidelines). We do not wish to replicate existing MHFA guidelines. Rather, our aim is to develop guidelines on how to tailor mental health first aid for a person assisting a co-worker who is experiencing mental health problem in the workplace.

Please rate how important (from 'essential' to 'should not be included') you think it is that each statement be included in the guidelines.

Please keep the definitions in mind when answering the questions.

Note: The following items relate to actions the person's MANAGER would carry out.

* 158. When approaching the person who has a substance use problem, the MANAGER should:

	Essential	Important	Don't know/Depends	Unimportant	Should not be included
Explain the effects that their substance use could have on co-workers and the workplace	○	○	○	○	○
Point out the possible workplace consequences for the person, e.g. loss of job.	○	○	○	○	○

159. Do you have any additional statements that you would like to add in this section? Please write your suggestions in the box provided.

How to help employees and co-workers with mental health problems

Thank you!

Thank you for your time!

That is the end of the first round survey. Thank you very much for your contribution.

If participating in this survey has caused you distress then we advise you to talk to a supportive person about these feelings. You might wish to talk to someone using the relevant helpline listed below.

Australia: Lifeline on 13 11 14

New Zealand: Lifeline Aotearoa on 0800 543 354

UK: Samaritans on 08457 909090

Republic of Ireland: Samaritans on 116 123

USA: National Suicide prevention Lifeline on 1800 273 TALK (8255)

Canada: National Suicide prevention Lifeline on 1800 273 TALK (8255)

By pressing the *'Next'* button your responses will be registered with our survey software. Once all panel members have lodged their responses, we will collate the data and send you a report on the findings and the second survey.

Workplace Delphi Round 2

1. Information about this survey

Purpose of the research

The aim of this current research project is to develop guidelines for how to provide mental health first aid to a co-worker. Once developed, these guidelines will provide guidance for employees on giving appropriate support to a co-worker who is developing a mental health problem or experiencing a mental health crisis.

The project is being conducted by Professor Tony Jorm, A/Prof Tony LaMontagne, A/Prof Angela Martin and Nataly Bovopoulos (PhD candidate) at the School of Population and Global Health, The University of Melbourne. This project has been approved by the University's Human Research Ethics Committee (HREC#: 1339508.1).

How this questionnaire was developed

The statements in this questionnaire were derived from the results of the Round 1 survey. You will note that each statement is marked as either a NEW or RERATE item. New items were derived from the comments provided in the first survey. An item is rerated when 70%–79% of panel members from all three groups rated it as essential or important.

What do I need to do?

Your task is to rate the statements presented in this questionnaire according to how important you believe they are to **providing mental health first aid to someone in the workplace**. When rating the statements, please keep in mind that the guidelines will be used by any employee in a workplace and as such, the statements need to be rated according to how important each one is for someone, who does not necessarily have a counselling or clinical background, to provide mental health first aid to another employee.

Note: In each section, there are some items that are actions for managers only.

For example you may be asked to rate the following statement:

1. The helper should not hurry the person because this can cause them anxiety.

You will need to decide how important, from *'essential'* to *'should not be included',* you think it is that this statement be included in the guidelines.

For an example of previously developed guidelines go to: First aid guidelines for assisting the person who is suicidal here.

Workplace Delphi Round 2

2. Information about this survey (cont)

How long will it take to complete the questionnaire?

This questionnaire should take approximately 30 minutes to complete.

Can I save my answers and come back to the questionnaire?

If necessary, you can complete the survey in two or more sittings. You can save your answers at any time by completing a page and clicking ‘Next’ at the bottom. This marks your page as complete and you can begin again at a later date on the next page. Please make sure that you always log back in using the **same computer,** and that cookies are enabled, otherwise the software will fail to recognise your code and previously saved responses.

Workplace Delphi Round 2

3. Overview of survey content

This survey is divided into the following sections:

1. General Awareness

2. Approach

3. Communication

4. Confidentiality

5. Distress

6. Support

7. Disclosure

8. Substance use problems and intoxication

9. Performance

10. Reasonable adjustments

Workplace Delphi Round 2

4. Definitions of terms used in this survey

These terms may have different meanings for participants. The definitions below are how these terms are used in this survey. When completing the survey, please ensure you use these definitions only.

Common mental health problems in this survey refer to signs and symptoms of problematic anxiety, depression or substance use.

Person refers to an employee with signs and symptoms of a common mental health problem.

Mental health first aid means the help provided by any employee to another employee who is developing a mental health problem or experiencing a mental health crisis. The help is provided until professional help is received or until the crisis resolves. This help may be provided by one person or by a range of different people.

Helper refers to any employee who provides mental health first aid as defined above. This helper does not necessarily have a designated role, such as a physical first aid officer has. The helper may be a co-worker of the person, be the person's manager, or in a role less senior to the person.

Workplace refers to a place of employment.

Employee refers to a person working for another person, business or organisation (who could also be working as a contractor or volunteer).

Manager refers to a person responsible for the supervision of one or more employees.

New term added: **Appropriate Manager** refers to the person's supervisor, HR, or the business owner depending on the size and nature of the workplace.

Workplace stressors refer to working conditions that can contribute to mental health problems, e.g., long working hours, excessive workload, poor supervisory support.

Reasonable adjustments refers to changes to the work environment that allow a person with a mental illness to work safely and productively.

Disclosure refers to the degree of information being shared with others at work about a person's mental health problem, e.g. functional limitations through to diagnosis.

Employee Assistance Programs refer to a free and confidential counselling service offered by employers to their employees to support their wellbeing in the workplace and in their personal lives.

Workplace Delphi Round 2

5. Section 1: General Awareness

This section contains statements about general awareness of mental health problems in the workplace.

There are already guidelines to help people who may be experiencing a range of mental health problems (click here to access these guidelines). We do not wish to replicate existing MHFA guidelines. Rather, our aim is to develop guidelines on how to tailor mental health first aid for a person assisting a co-worker who is experiencing mental health problem in the workplace.

Please rate how important (from 'essential' to 'should not be included') you think it is that each statement be included in the guidelines.

Please keep the definitions in mind when answering the questions.

* 1. The helper should be aware of the ways in which mental health problems may make a person a better employee, e.g. more aware of their own strengths and weaknesses, more empathic towards co-workers. (RERATE)

Essential	Important	Don't know/ Depends	Unimportant	Should not be included
○	○	○	○	○

* 2. If the helper is aware that the person has a history of mental health problems, they should not assume that their moods or behaviours are necessarily due to these problems. (NEW)

Essential	Important	Don't know/Depends	Unimportant	Should not be included
○	○	○	○	○

* 3. The helper should not normalise problem behaviours as part of the person's personality, as this may make it difficult to recognise mental health problems. (RERATE)

Essential	Important	Don't know/Depends	Unimportant	Should not be included
○	○	○	○	○

Workplace Delphi Round 2

6. Section 2: Approach

This section contains statements about approaching the person to talk about their concerns.

There are already guidelines to help people who may be experiencing a range of mental health problems (click here to access these guidelines). We do not wish to replicate existing MHFA guidelines. Rather, our aim is to develop guidelines on how to tailor mental health first aid for a person assisting a co-worker who is experiencing mental health problem in the workplace.

Please rate how important (from 'essential' to 'should not be included') you think it is that each statement be included in the guidelines.

Please keep the definitions in mind when answering the questions.

Deciding whether to approach the person

* 4. If the helper notices worrying changes in the person's work or interactions with others, they should discuss this with them. (RERATE)

Essential	Important	Don't know/Depends	Unimportant	Should not be included
○	○	○	○	○

* 5. If the person is showing signs or symptoms of mental health problems, the helper should offer assistance, even if the person is still working productively. (RERATE)

Essential	Important	Don't know/Depends	Unimportant	Should not be included
○	○	○	○	○

* 6. The helper should approach the person showing signs of a mental health problem, even if the person is still working productively. (NEW)

Essential	Important	Don't know/Depends	Unimportant	Should not be included
○	○	○	○	○

* 7. The helper should consider the following in deciding whether to approach the person:

	Essential	Important	Don't know/ Depends	Unimportant	Should not be included
If they are the person's manager, whether there is a conflict between their management and first aid roles (RERATE)	○	○	○	○	○
If they are the person's manager, and feel that they may be contributing to the person's mental health problems (NEW)	○	○	○	○	○
Whether the workplace culture is contributing to the person's mental health problem. (RERATE)	○	○	○	○	○

Workplace Delphi Round 2

7. Section 2: Approach (cont)

This section contains statements about approaching the person to talk about their concerns.

There are already guidelines to help people who may be experiencing a range of mental health problems (click here to access these guidelines). We do not wish to replicate existing MHFA guidelines. Rather, our aim is to develop guidelines on how to tailor mental health first aid for a person assisting a co-worker who is experiencing mental health problem in the workplace.

Please rate how important (from ‘essential’ to ‘should not be included’) you think it is that each statement be included in the guidelines.

Please keep the definitions in mind when answering the questions.

Involving other co-workers

* 8. If there is no one else available or appropriate to help, the helper should:

	Essential	Important	Don't know/ Depends	Unimportant	Should not be included
Talk about the situation with someone else who can advise on how to approach the person, while keeping the person's identity anonymous. (NEW)	○	○	○	○	○
Talk with a mental health professional about how to approach the person. (RERATE)	○	○	○	○	○

* 9. If the helper seeks advice from someone else, they should emphasise the importance of maintaining the person's privacy and confidentiality. (NEW)

Essential	Important	Don't know/Depends	Unimportant	Should not be included
○	○	○	○	○

* 10. If the person is in a role that is senior to the helper and they do not feel they can make the approach, they should consider:

	Essential	Important	Don't know/ Depends	Unimportant	Should not be included
Contacting an impartial, external source of support (e.g. Employee Assistance Program, helpline) for advice (NEW)	○	○	○	○	○
Contacting a telephone support service for advice e.g. Lifeline (NEW)	○	○	○	○	○
Identifying an appropriate helper within the organisation and contacting them (NEW)	○	○	○	○	○
Speaking to someone else who has a good relationship with the person (NEW)	○	○	○	○	○
Whether approaching the person will have negative consequences to the helper's career. (NEW)	○	○	○	○	○

* 11. If the helper is uncertain whether the person has mental health problems, the helper should **not** ask other co-workers whether they have observed any concerning behaviours. (NEW)

Essential	Important	Don't know/Depends	Unimportant	Should not be included
○	○	○	○	○

* 12. The helper should **not** discuss their concerns with other employees who may be affected by the person's behaviour, such as other team members or managers. (NEW)

Essential	Important	Don't know/Depends	Unimportant	Should not be included
○	○	○	○	○

* 13. If the person's behaviour is affecting others in the workplace and the person's manager has appropriate training, the helper should approach this manager. (NEW)

Essential	Important	Don't know/Depends	Unimportant	Should not be included
○	○	○	○	○

* 14. If after following up with the manager the concerns are still not addressed satisfactorily, the helper should raise their concerns with:

	Essential	Important	Don't know/ Depends	Unimportant	Should not be included
HR (NEW)	○	○	○	○	○
Employee Assistance Program (NEW)	○	○	○	○	○
The health and safety manager or representative. (NEW)	○	○	○	○	○

Workplace Delphi Round 2

8. Section 2: Approach (cont)

This section contains statements about approaching the person to talk about their concerns.

There are already guidelines to help people who may be experiencing a range of mental health problems (click here to access these guidelines). We do not wish to replicate existing MHFA guidelines. Rather, our aim is to develop guidelines on how to tailor mental health first aid for a person assisting a co-worker who is experiencing mental health problem in the workplace.

Please rate how important (from 'essential' to 'should not be included') you think it is that each statement be included in the guidelines.

Please keep the definitions in mind when answering the questions.

Planning the approach

* 15. Before the helper approaches the person, they should be clear about concerning behaviours they have noticed. (NEW)

Essential	Important	Don't know/Depends	Unimportant	Should not be included
○	○	○	○	○

* 16. Before the helper approaches the person, they should be clear about how the concerning behaviours are affecting them. (NEW)

Essential	Important	Don't know/Depends	Unimportant	Should not be included
○	○	○	○	○

* 17. The helper should consider approaching the person by having a casual conversation and asking whether they are OK. (NEW)

Essential	Important	Don't know/Depends	Unimportant	Should not be included
○	○	○	○	○

* 18. When approaching the person, the helper should consider a staggered approach, e.g. starting with a casual approach asking how they are, followed by a meeting if appropriate. (NEW)

Essential	Important	Don't know/Depends	Unimportant	Should not be included
○	○	○	○	○

* 19. The helper should have the conversation with the person in a neutral private space, such as outside the office in a cafe, or somewhere where the person feels comfortable and conversations can't be overheard. (RERATE)

Essential	Important	Don't know/Depends	Unimportant	Should not be included
○	○	○	○	○

* 20. The helper should have the conversation with the person in an appropriate neutral private space. (NEW)

Essential	Important	Don't know/Depends	Unimportant	Should not be included
○	○	○	○	○

* 21. The helper should give the person the option of meeting in ways other than face-to-face, e.g. online chat, phone. (NEW)

Essential	Important	Don't know/Depends	Unimportant	Should not be included
○	○	○	○	○

Workplace Delphi Round 2

9. Section 2: Approach (cont)

This section contains statements about approaching the person to talk about their concerns.

There are already guidelines to help people who may be experiencing a range of mental health problems (click here to access these guidelines). We do not wish to replicate existing MHFA guidelines. Rather, our aim is to develop guidelines on how to tailor mental health first aid for a person assisting a co-worker who is experiencing mental health problem in the workplace.

Please rate how important (from ‘essential’ to ‘should not be included’) you think it is that each statement be included in the guidelines.

Please keep the definitions in mind when answering the questions.

How the person's MANAGER would make the approach

* 22. Before approaching the person, the MANAGER should:

	Essential	Important	Don't know/ Depends	Unimportant	Should not be included
Document any concerning behaviours that they have observed. (NEW)					
Have on hand any documentation relating to work performance or observed behaviours. (NEW)					
Consider the expected outcomes and plan the structure and aims of the discussion in advance. (NEW)					
Be clear about how the concerning behaviours are affecting others. (NEW)					
Check any relevant organisational policies and procedures regarding performance problems if the person does not disclose a mental health problem. (RERATE)					

* 23. When approaching the person, the MANAGER should consider:

	Essential	Important	Don't know/ Depends	Unimportant	Should not be included
Going to the person's worksite if they are a remote worker. (RERATE)					
Alternate ways of meeting if they are a remote worker, e.g. video conferencing, online chat. (NEW)					

* 24. If the MANAGER wishes to approach a person who works offsite, they should organise the visit ahead of time with the person, rather than doing it unannounced. (NEW)

Essential	Important	Don't know/Depends	Unimportant	Should not be included

* 25. When approaching the person, the MANAGER should emphasise their concern for the person's wellbeing rather than their work performance. (NEW)

Essential	Important	Don't know/Depends	Unimportant	Should not be included

* 26. When talking with the person about their concerns, the MANAGER should:

	Essential	Important	Don't know/ Depends	Unimportant	Should not be included
First talk about the person's strengths and how they are valued. (NEW)	◯	◯	◯	◯	◯
Stay focused on work-related issues. (NEW)	◯	◯	◯	◯	◯
Focus the discussion on the person's health and wellbeing, rather than their work performance. (NEW)	◯	◯	◯	◯	◯
Ask open-ended questions about how any mental health problems may be affecting the person. (NEW)	◯	◯	◯	◯	◯
Be honest and open when the person's performance is being affected, even if they are worried that this may distress the person. (NEW)	◯	◯	◯	◯	◯

* 27. After raising their concerns with the person, the MANAGER should ask:

	Essential	Important	Don't know/ Depends	Unimportant	Should not be included
If the person would like to continue the conversation in the presence of a support person, e.g. an external advocate, co-worker (NEW)	◯	◯	◯	◯	◯
What impact any mental health problems are having on them (NEW)	◯	◯	◯	◯	◯
How long they have been experiencing a mental health problem, to determine whether this is an ongoing issue or something where an immediate action could assist the situation (NEW)	◯	◯	◯	◯	◯
Whether the person has ever been given a diagnosis of a mental illness (NEW)	◯	◯	◯	◯	◯
If they have an advance directive or management plan in the workplace, if the person discloses a mental illness (NEW)	◯	◯	◯	◯	◯
Whether any workplace issues are contributing to their mental health problems (NEW)	◯	◯	◯	◯	◯
Whether any non-workplace issues are contributing to their mental health problems. (NEW)	◯	◯	◯	◯	◯

* 28. If the MANAGER's expectations of the discussion are not met, they should try to speak to the person again at another time. (NEW)

Essential	Important	Don't know/Depends	Unimportant	Should not be included
◯	◯	◯	◯	◯

Workplace Delphi Round 2

10. Section 3: Communication

This section contains statements about communicating with the person in the workplace.

There are already guidelines to help people who may be experiencing a range of mental health problems (click here to access these guidelines). We do not wish to replicate existing MHFA guidelines. Rather, our aim is to develop guidelines on how to tailor mental health first aid for a person assisting a co-worker who is experiencing mental health problem in the workplace.

Please rate how important (from ‘essential’ to ‘should not be included’) you think it is that each statement be included in the guidelines.

Please keep the definitions in mind when answering the questions.

* 29. If while talking with the person, the helper decides that they are not the right person to help, they should:

	Essential	Important	Don't know/ Depends	Unimportant	Should not be included
Tell the person. (NEW)	○	○	○	○	○
Discuss with the person alternative sources of help. (NEW)	○	○	○	○	○
Offer to seek the immediate assistance of someone with appropriate training. (NEW)	○	○	○	○	○

* 30. The helper should create a supportive and trusting environment when discussing mental health problems with the person by:

	Essential	Important	Don't know/ Depends	Unimportant	Should not be included
Sitting an appropriate distance away, taking into account personal space (RERATE)	○	○	○	○	○
Considering what they know about the person and their culture. (NEW)	○	○	○	○	○

Workplace Delphi Round 2

11. Section 3: Communication (cont)

This section contains statements about communicating with the person in the workplace.

There are already guidelines to help people who may be experiencing a range of mental health problems (click here to access these guidelines). We do not wish to replicate existing MHFA guidelines. Rather, our aim is to develop guidelines on how to tailor mental health first aid for a person assisting a co-worker who is experiencing mental health problem in the workplace.

Please rate how important (from ‘essential’ to ‘should not be included’) you think it is that each statement be included in the guidelines.

Please keep the definitions in mind when answering the questions.

What to discuss

* 31. When talking with the person about their concerns, the helper should:

	Essential	Important	Don't know/ Depends	Unimportant	Should not be included
Focus the discussion on the person's health and wellbeing, rather than their work performance. (RERATE)	○	○	○	○	○
Ask open-ended questions about how any mental health problems may be affecting the person. (RERATE)	○	○	○	○	○
Consider sharing any personal experiences of mental health problems with the person that are relevant to the conversation. (NEW)	○	○	○	○	○

* 32. When talking with the person, the helper should say what feels comfortable for them. (RERATE)

Essential	Important	Don't know/ Depends	Unimportant	Should not be included
○	○	○	○	○

* 33. After raising their concerns with the person, the helper should ask, if the person would like to continue the conversation in the presence of a support person, e.g. an external advocate, co-worker. (RERATE)

Essential	Important	Don't know/ Depends	Unimportant	Should not be included
○	○	○	○	○

* 34. The helper should ask the person:

	Essential	Important	Don't know/ Depends	Unimportant	Should not be included
If there are any non-work related issues they may like to talk about or make the helper aware of. (RERATE)	○	○	○	○	○
Whether any *workplace or other* issues are contributing to their mental health problems. (RERATE)	○	○	○	○	○
Whether any *workplace* issues are contributing to their mental health problems. (NEW)	○	○	○	○	○
Whether any *non-workplace* issues are contributing to their mental health problems. (NEW)	○	○	○	○	○

* 35. If the person says that workplace stressors are contributing to their mental health problem, the helper should encourage the person to talk to an appropriate manager about this. (NEW)

Essential	Important	Don't know/ Depends	Unimportant	Should not be included
○	○	○	○	○

* 36. If the person says that workplace stressors are contributing to their mental health problem, the helper should offer to assist them to find appropriate information and support. (NEW)

Essential	Important	Don't know/ Depends	Unimportant	Should not be included
○	○	○	○	○

Workplace Delphi Round 2

12. Section 3: Communication (cont)

This section contains statements about communicating with the person in the workplace.

There are already guidelines to help people who may be experiencing a range of mental health problems (click here to access these guidelines). We do not wish to replicate existing MHFA guidelines. Rather, our aim is to develop guidelines on how to tailor mental health first aid for a person assisting a co-worker who is experiencing mental health problem in the workplace.

Please rate how important (from 'essential' to 'should not be included') you think it is that each statement be included in the guidelines.

Please keep the definitions in mind when answering the questions.

Things to avoid

* 37. The helper should **not** start the conversation with the person by talking about how their own personal struggles have affected their work behaviour. (NEW)

Essential	Important	Don't know/Depends	Unimportant	Should not be included
○	○	○	○	○

* 38. The helper should **not** have documentation about the person on hand when meeting with them. (NEW)

Essential	Important	Don't know/Depends	Unimportant	Should not be included
○	○	○	○	○

* 39. The helper should **not** take notes while meeting with the person. (NEW)

Essential	Important	Don't know/Depends	Unimportant	Should not be included
○	○	○	○	○

* 40. The helper should not:

	Essential	Important	Don't know/ Depends	Unimportant	Should not be included
Counsel the person (RERATE)					
Try to act as a counsellor (NEW)					
Adopt an overly friendly attitude (RERATE)					

Workplace Delphi Round 2

13. Section 3: Communication (cont)

This section contains statements about communicating with the person in the workplace.

There are already guidelines to help people who may be experiencing a range of mental health problems (click here to access these guidelines). We do not wish to replicate existing MHFA guidelines. Rather, our aim is to develop guidelines on how to tailor mental health first aid for a person assisting a co-worker who is experiencing mental health problem in the workplace.

Please rate how important (from 'essential' to 'should not be included') you think it is that each statement be included in the guidelines.

Please keep the definitions in mind when answering the questions.

If they don't want to talk

* 41. If the person chooses not to talk, the helper should:

	Essential	Important	Don't know/ Depends	Unimportant	Should not be included
Ask them if they would find it helpful if they stayed with the person without needing to talk. (NEW)					
Provide information for the person to take away and look at later. (NEW)					
Touch base with the person at a later time to see if they are more willing to talk. (NEW)					

Workplace Delphi Round 2

14. Section 3: Communication (cont)

This section contains statements about communicating with the person in the workplace.

There are already guidelines to help people who may be experiencing a range of mental health problems (click here to access these guidelines). We do not wish to replicate existing MHFA guidelines. Rather, our aim is to develop guidelines on how to tailor mental health first aid for a person assisting a co-worker who is experiencing mental health problem in the workplace.

Please rate how important (from 'essential' to 'should not be included') you think it is that each statement be included in the guidelines.

Please keep the definitions in mind when answering the questions.

Communicating with other workers

* 42. If the person has chosen **not** to disclose their mental health issues to other co-workers, the helper should refer any inquiries or complaints about the person to the person's manager. (NEW)

Essential	Important	Don't know/Depends	Unimportant	Should not be included
○	○	○	○	○

* 43. If the person has chosen **not** to disclose their mental health issues to co-workers, the MANAGER should only speak with co-workers about the person in general terms without breaching confidentiality. (NEW)

Essential	Important	Don't know/Depends	Unimportant	Should not be included
○	○	○	○	○

* 44. If the helper hears other employees talking about the details of the person's mental health problems in an unhelpful way, they should let an appropriate manager know. (NEW)

Essential	Important	Don't know/Depends	Unimportant	Should not be included
○	○	○	○	○

Workplace Delphi Round 2

15. Section 4: Confidentiality

This section contains statements about dealing with confidentiality issues with the person in the workplace.

There are already guidelines to help people who may be experiencing a range of mental health problems (click here to access these guidelines). We do not wish to replicate existing MHFA guidelines. Rather, our aim is to develop guidelines on how to tailor mental health first aid for a person assisting a co-worker who is experiencing mental health problem in the workplace.

Please rate how important (from 'essential' to 'should not be included') you think it is that each statement be included in the guidelines.

Please keep the definitions in mind when answering the questions.

* 45. The helper should be clear with the person about the limits of confidentiality early in the conversation. (NEW)

Essential	Important	Don't know/Depends	Unimportant	Should not be included
○	○	○	○	○

* 46. The MANAGER should be clear with the person about the limits of confidentiality early in the conversation. (NEW)

Essential	Important	Don't know/Depends	Unimportant	Should not be included
○	○	○	○	○

* 47. The MANAGER should maintain the person's confidentiality and privacy except where there is a concern:

	Essential	Important	Don't know/ Depends	Unimportant	Should not be included
For harm to the organisation, e.g. reputational harm (NEW)	○	○	○	○	○
That the person cannot fulfil the inherent requirements of the role. (NEW)	○	○	○	○	○

Workplace Delphi Round 2

16. Section 5: Distress

This section contains statements about supporting the person who is distressed in the workplace.

There are already guidelines to help people who may be experiencing a range of mental health problems (click here to access these guidelines). We do not wish to replicate existing MHFA guidelines. Rather, our aim is to develop guidelines on how to tailor mental health first aid for a person assisting a co-worker who is experiencing mental health problem in the workplace.

Please rate how important (from ‘essential’ to ‘should not be included’) you think it is that each statement be included in the guidelines.

Please keep the definitions in mind when answering the questions.

* 48. If the person is distressed, the helper should do the following (where appropriate to the circumstances and the helper's work role):

	Essential	Important	Don't know/ Depends	Unimportant	Should not be included
Not send the person home straight away (RERATE)	◯	◯	◯	◯	◯
Let them know that once they have recovered sufficiently, they can carry on working, take a break, or seek permission to go home. (RERATE)	◯	◯	◯	◯	◯
Remember that the person may be comforted by just having someone there to listen to them. (NEW)	◯	◯	◯	◯	◯
Ring the EAP (if available), with the person's permission, and let the person speak to them. (NEW)	◯	◯	◯	◯	◯
Acknowledge the person's distress rather than immediately try to problem solve. (NEW)	◯	◯	◯	◯	◯

* 49. If the person is distressed *and reluctant to talk*, the helper should (where appropriate to the circumstances and the helper's work role), ask the person if they wish to communicate in another way such as online chat or email. (NEW)

Essential	Important	Don't know/ Depends	Unimportant	Should not be included
◯	◯	◯	◯	◯

Workplace Delphi Round 2

17. Section 6: Support

This section contains statements about providing support to the person.

There are already guidelines to help people who may be experiencing a range of mental health problems (click here to access these guidelines). We do not wish to replicate existing MHFA guidelines. Rather, our aim is to develop guidelines on how to tailor mental health first aid for a person assisting a co-worker who is experiencing mental health problem in the workplace.

Please rate how important (from ‘essential’ to ‘should not be included’) you think it is that each statement be included in the guidelines.

Please keep the definitions in mind when answering the questions.

* 50. The helper should ask about supports outside of work that the person may find helpful. (NEW)

Essential	Important	Don't know/Depends	Unimportant	Should not be included
◯	◯	◯	◯	◯

* 51. If the person does not initially know what support they need, the helper should assist them in exploring the available options. (RERATE)

Essential	Important	Don't know/Depends	Unimportant	Should not be included
○	○	○	○	○

* 52. The helper should encourage the person to consider talking to their manager about their concerns. (RERATE)

Essential	Important	Don't know/ Depends	Unimportant	Should not be included
○	○	○	○	○

* 53. If the person has an advance directive or management plan, the helper should:

	Essential	Important	Don't know/ Depends	Unimportant	Should not be included
Follow the instructions (RERATE)	○	○	○	○	○
Seek assistance from an appropriate manager in order to follow it. (NEW)	○	○	○	○	○

* 54. If the person discloses a mental illness and does not have an advance directive or management plan, the helper should suggest they develop one. (NEW)

Essential	Important	Don't know/Depends	Unimportant	Should not be included
○	○	○	○	○

* 55. The helper should **not** encourage the person to consider leaving their job, even if work-related stressors continue to contribute to their mental health problems. (NEW)

Essential	Important	Don't know/Depends	Unimportant	Should not be included
○	○	○	○	○

* 56. If the person agrees, the helper should check in with the person regularly in order to provide ongoing support and encouragement. (NEW)

Essential	Important	Don't know/Depends	Unimportant	Should not be included
○	○	○	○	○

Workplace Delphi Round 2

18. Section 6: Support (cont.)

This section contains statements about providing support to the person.

There are already guidelines to help people who may be experiencing a range of mental health problems (click here to access these guidelines). We do not wish to replicate existing MHFA guidelines. Rather, our aim is to develop guidelines on how to tailor mental health first aid for a person assisting a co-worker who is experiencing mental health problem in the workplace.

Please rate how important (from 'essential' to 'should not be included') you think it is that each statement be included in the guidelines.

Please keep the definitions in mind when answering the questions.

Note: The following items relate to how the person's MANAGER would provide support to the person.

* 57. The MANAGER should encourage co-workers to support the person, whilst still respecting the person's privacy. (RERATE)

Essential	Important	Don't know/Depends	Unimportant	Should not be included
○	○	○	○	○

* 58. The MANAGER should ask the person's permission to encourage co-workers to support the person, whilst still respecting the person's privacy. (NEW)

Essential	Important	Don't know/Depends	Unimportant	Should not be included
○	○	○	○	○

* 59. The MANAGER should tell the person that they will not tolerate any discrimination towards the person at work due to their mental health problems. (NEW)

Essential	Important	Don't know/Depends	Unimportant	Should not be included
○	○	○	○	○

* 60. The MANAGER should tell the person that they will help them get the support they need to return to their usual work performance. (RERATE)

Essential	Important	Don't know/Depends	Unimportant	Should not be included
○	○	○	○	○

* 61. The MANAGER should tell the person that they will help them get the support they need *that will assist them* in returning to their usual work performance. (NEW)

Essential	Important	Don't know/Depends	Unimportant	Should not be included
○	○	○	○	○

* 62. The MANAGER should assist the person to consider the pros and cons of taking time off work. (NEW)

Essential	Important	Don't know/Depends	Unimportant	Should not be included
○	○	○	○	○

* 63. If the person is able to meet the majority of their job requirements, the MANAGER should encourage them to keep coming to work. (NEW)

Essential	Important	Don't know/Depends	Unimportant	Should not be included
◯	◯	◯	◯	◯

* 64. The MANAGER should encourage the appropriate use of sick leave entitlements. (RERATE)

Essential	Important	Don't know/Depends	Unimportant	Should not be included
◯	◯	◯	◯	◯

* 65. The MANAGER should inform the person of the appropriate use of sick leave entitlements. (NEW)

Essential	Important	Don't know/Depends	Unimportant	Should not be included
◯	◯	◯	◯	◯

* 66. The MANAGER should assist the person to identify workplace triggers that contribute to their mental health problems. (NEW)

Essential	Important	Don't know/Depends	Unimportant	Should not be included
◯	◯	◯	◯	◯

* 67. The MANAGER should check in with the person regularly in order to provide ongoing support and encouragement. (NEW)

Essential	Important	Don't know/Depends	Unimportant	Should not be included
◯	◯	◯	◯	◯

* 68. *If the person agrees*, the MANAGER should check in with them regularly in order to provide ongoing support and encouragement. (NEW)

Essential	Important	Don't know/Depends	Unimportant	Should not be included
◯	◯	◯	◯	◯

Workplace Delphi Round 2

19. Section 7: Disclosure

This section contains statements about the person disclosing their mental health problem in the workplace.

There are already guidelines to help people who may be experiencing a range of mental health problems (click here to access these guidelines). We do not wish to replicate existing MHFA guidelines. Rather, our aim is to develop guidelines on how to tailor mental health first aid for a person assisting a co-worker who is experiencing mental health problem in the workplace.

Please rate how important (from ‘essential’ to ‘should not be included’) you think it is that each statement be included in the guidelines.

Please keep the definitions in mind when answering the questions.

* 69. The helper should be aware of any relevant discrimination legislation in relation to the disclosure of mental health problems at work. (RERATE)

Essential	Important	Don't know/Depends	Unimportant	Should not be included
○	○	○	○	○

* 70. *If the person discloses that they have a mental health problem,* the helper should ask them:

	Essential	Important	Don't know/ Depends	Unimportant	Should not be included
What impact the mental health problems are having on them (NEW)	○	○	○	○	○
How long they been experiencing a mental health problem, to determine whether this is an ongoing issue or something where an immediate action could assist the situation (NEW)	○	○	○	○	○
Whether the person has ever been given a diagnosis of a mental illness (NEW)	○	○	○	○	○
If they have an advance directive or management plan in the workplace in case of an episode, if the person discloses a diagnosis of a mental illness (NEW)	○	○	○	○	○
If there are any non-work related issues they may like to talk about or make the helper aware of (NEW)	○	○	○	○	○
Whether any workplace or other issues are contributing to their mental health problems. (NEW)	○	○	○	○	○

* 71. The helper should discuss with the person the issues around disclosing their mental health problems to *co-workers*. (NEW)

Essential	Important	Don't know/Depends	Unimportant	Should not be included
○	○	○	○	○

* 72. The helper should discuss with the person the issues around disclosing their mental health problems to *their manager*. (NEW)

Essential	Important	Don't know/Depends	Unimportant	Should not be included
○	○	○	○	○

* 73. The helper should assist the person to consider the pros and cons of disclosing mental health problems to *co-workers.* (NEW)

Essential	Important	Don't know/Depends	Unimportant	Should not be included
○	○	○	○	○

* 74. The helper should assist the person to consider the pros and cons of disclosing mental health problems to *their manager.* (NEW)

Essential	Important	Don't know/Depends	Unimportant	Should not be included
○	○	○	○	○

* 75. The helper should **not** ask the person if they are receiving any treatment for mental health problems unless the person discloses a mental health problem. (NEW)

Essential	Important	Don't know/Depends	Unimportant	Should not be included
○	○	○	○	○

Workplace Delphi Round 2

20. Section 7: Disclosure (cont)

This section contains statements about the person disclosing their mental health problem in the workplace.

There are already guidelines to help people who may be experiencing a range of mental health problems (click here to access these guidelines). We do not wish to replicate existing MHFA guidelines. Rather, our aim is to develop guidelines on how to tailor mental health first aid for a person assisting a co-worker who is experiencing mental health problem in the workplace.

Please rate how important (from ‘essential’ to ‘should not be included’) you think it is that each statement be included in the guidelines.

Please keep the definitions in mind when answering the questions.

Note: The following items relate to how the person’s MANAGER would approach disclosure issues.

* 76. The MANAGER should be aware of any legal obligations that the person has to disclose that they have a mental health problem at work, e.g. medical practitioners may be required to report medical conditions that impair their performance. (NEW)

Essential	Important	Don't know/Depends	Unimportant	Should not be included
○	○	○	○	○

* 77. The MANAGER should be aware of any relevant discrimination legislation in relation to the disclosure of mental health problems at work. (NEW)

Essential	Important	Don't know/Depends	Unimportant	Should not be included
○	○	○	○	○

Workplace Delphi Round 2

21. Section 8: Substance use problems and intoxication

This section is about helping the person who has a substance use problem or is intoxicated in the workplace.

There are already guidelines to help people who may be experiencing a range of mental health problems (click here to access these guidelines). We do not wish to replicate existing MHFA guidelines. Rather, our aim is to develop guidelines on how to tailor mental health first aid for a person assisting a co-worker who is experiencing mental health problem in the workplace.

Please rate how important (from 'essential' to 'should not be included') you think it is that each statement be included in the guidelines.

Please keep the definitions in mind when answering the questions.

* 78. If the person appears to be affected by drugs or alcohol while at work, the helper should:

	Essential	Important	Don't know/ Depends	Unimportant	Should not be included
Speak to an appropriate manager. (NEW)	○	○	○	○	○
Call in a more senior co-worker to serve as a witness if possible. (RERATE)	○	○	○	○	○
Inform the person of relevant policies that cover substance use at work. (NEW)	○	○	○	○	○
Ask the person for any explanation for their behaviour, e.g. side-effects of a medication. (RERATE)	○	○	○	○	○
Arrange for the person to be escorted home safely. (RERATE)	○	○	○	○	○
Notify an appropriate authority or emergency contact if the person insists on driving home. (NEW)	○	○	○	○	○

* 79. If the person appears to be affected by drugs or alcohol while at work, the MANAGER should:

	Essential	Important	Don't know/ Depends	Unimportant	Should not be included
Ask the person for any explanation for their behaviour, e.g. side-effects of a medication. (NEW)	○	○	○	○	○
Arrange for the person to be escorted home safely. (NEW)	○	○	○	○	○
Notify an appropriate authority or emergency contact if the person insists on driving home. (NEW)	○	○	○	○	○
Notify the police if the person insists on driving home. (NEW)	○	○	○	○	○
Involve the health and safety representative. (NEW)	○	○	○	○	○

* 80. If the person uses substances outside of work hours and it is affecting their functioning in some negative way, the helper should approach the person about this, even if their work performance is not affected. (NEW)

Essential	Important	Don't know/Depends	Unimportant	Should not be included
○	○	○	○	○

* 81. If the person uses substances outside of work hours, the MANAGER should only approach the person about this if it impacts on the person's work performance. (NEW)

Essential	Important	Don't know/Depends	Unimportant	Should not be included
○	○	○	○	○

* 82. If the person uses substances outside of work hours and this does not affect the person's work performance, the MANAGER should still approach the person to let them know that they are concerned about their health. (NEW)

Essential	Important	Don't know/Depends	Unimportant	Should not be included
○	○	○	○	○

* 83. The helper should **not** organise a team meeting to confront the person, even if all other attempts to help the person who is misusing substances fail. (NEW)

Essential	Important	Don't know/Depends	Unimportant	Should not be included
○	○	○	○	○

Workplace Delphi Round 2

22. Section 9: Performance

This section is about how the person's MANAGER should give mental health first aid to the person who also requires performance management.

There are already guidelines to help people who may be experiencing a range of mental health problems (click here to access these guidelines). We do not wish to replicate existing MHFA guidelines. Rather, our aim is to develop guidelines on how to tailor mental health first aid for a person assisting a co-worker who is experiencing mental health problem in the workplace.

Please rate how important (from 'essential' to 'should not be included') you think it is that each statement be included in the guidelines.

Please keep the definitions in mind when answering the questions.

* 84. If the MANAGER is engaging in performance management processes, they should also encourage the person to seek help for any mental health problems. (NEW)

Essential	Important	Don't know/Depends	Unimportant	Should not be included
○	○	○	○	○

* 85. The MANAGER should focus on providing appropriate support for the person's mental health problems, before they address any performance issues. (NEW)

Essential	Important	Don't know/Depends	Unimportant	Should not be included
○	○	○	○	○

* 86. The MANAGER should focus on encouraging help-seeking, before they address any performance issues. (RERATE)

Essential	Important	Don't know/Depends	Unimportant	Should not be included
○	○	○	○	○

* 87. The MANAGER should focus on performance management only after an agreed period of reasonable adjustments and after encouraging the person to seek professional help. (RERATE)

Essential	Important	Don't know/Depends	Unimportant	Should not be included
○	○	○	○	○

* 88. When the MANAGER is providing support for the person's mental health problems and is required to initiate performance management processes, they should:

	Essential	Important	Don't know/ Depends	Unimportant	Should not be included
Be specific about the purpose of any meeting. (NEW)	○	○	○	○	○
Ask the person if they would like a support person present. (NEW)	○	○	○	○	○

* 89. If at any point it becomes apparent that the person's behaviours are breaching the workplace's rules or regulations, the MANAGER should end the conversation and explain that a formal meeting will need to be arranged. (NEW)

Essential	Important	Don't know/Depends	Unimportant	Should not be included
○	○	○	○	○

Workplace Delphi Round 2

23. Section 10: Reasonable Adjustments

This section contains statements about reasonable adjustments and the person.

There are already guidelines to help people who may be experiencing a range of mental health problems (click here to access these guidelines). We do not wish to replicate existing MHFA guidelines. Rather, our aim is to develop guidelines on how to tailor mental health first aid for a person assisting a co-worker who is experiencing mental health problem in the workplace.

Please rate how important (from 'essential' to 'should not be included') you think it is that each statement be included in the guidelines.

Please keep the definitions in mind when answering the questions.

* 90. The helper should be able to explain the concept of 'reasonable adjustments' to the person. (NEW)

Essential	Important	Don't know/Depends	Unimportant	Should not be included
○	○	○	○	○

* 91. If the person is having difficulties with their performance because of their mental health problems, the helper should suggest they speak to an appropriate manager about reasonable adjustments. (NEW)

Essential	Important	Don't know/Depends	Unimportant	Should not be included
○	○	○	○	○

* 92. If the organisation has no policies for accommodating employees with mental health problems, the MANAGER should seek advice from an appropriate source, e.g. HR, EAP. (NEW)

Essential	Important	Don't know/Depends	Unimportant	Should not be included
○	○	○	○	○

* 93. If the person is having difficulties with their performance because of their mental health problems, the MANAGER should discuss reasonable adjustments with them. (NEW)

Essential	Important	Don't know/Depends	Unimportant	Should not be included
○	○	○	○	○

Workplace Delphi Round 2

24. Thank you!

Thank you for your time!

That is the end of the Round 2 survey. Thank you very much for your contribution.

If participating in this survey has caused you distress then we advise you to talk to a supportive person about these feelings. You might wish to talk to someone using the relevant helpline listed below.

Australia: Lifeline on 13 11 14

New Zealand: Lifeline Aotearoa on 0800 543 354

UK: Samaritans on 08457 909090

Republic of Ireland: Samaritans on 116 123

USA: National Suicide prevention Lifeline on 1800 273 TALK (8255)

Canada: National Suicide prevention Lifeline on 1800 273 TALK (8255)

By pressing the *'Next'* button your responses will be registered with our survey software. Once all panel members have lodged their responses, we will collate the data and send you a report on the findings and the third and final survey.

Workplace Delphi Round 3

1. Information about this survey

Purpose of the research

The aim of this current research project is to develop guidelines for how to provide mental health first aid to a co-worker. Once developed, these guidelines will provide guidance for employees on giving appropriate support to a co-worker who is developing a mental health problem or experiencing a mental health crisis.

The project is being conducted by Professor Tony Jorm, A/Prof Tony LaMontagne, A/Prof Angela Martin and Nataly Bovopoulos (PhD candidate) at the School of Population and Global Health, The University of Melbourne. This project has been approved by the University's Human Research Ethics Committee (HREC#: 1339508.1).

How this questionnaire was developed

The statements in this questionnaire were derived from the results of the Round 2 survey. Only statements that need to be rerated are included in this survey. An item is rerated when 70%–79% of panel members from all three groups rated it as essential or important.

What do I need to do?

Your task is to rate the statements presented in this questionnaire according to how important you believe they are to providing mental health first aid to someone in the workplace. When rating the statements, please keep in mind that the guidelines will be used by any employee in a workplace and as such, the statements need to be rated according to how important each one is for someone, who does not necessarily have a counselling or clinical background, to provide mental health first aid to another employee. Note: In each section, there are some items that are actions for managers only.

For an example of previously developed guidelines go to: First aid guidelines for assisting the person who is suicidal here.

How long will it take to complete the questionnaire?

This questionnaire should take approximately 10 minutes to complete.

Can I save my answers and come back to the questionnaire?

If necessary, you can complete the survey in two or more sittings. You can save your answers at any time by completing a page and clicking 'Next' at the bottom. This marks your page as complete and you can begin again at a later date on the next page. Please make sure that you always log back in using the same computer, and that cookies are enabled, otherwise the software will fail to recognise your code and previously saved responses.

Workplace Delphi Round 3

2. Definitions of terms used in this survey

These terms may have different meanings for participants. The definitions below are how these terms are used in this survey. When completing the survey, please ensure you use these definitions only.

Common mental health problems in this survey refer to signs and symptoms of problematic anxiety, depression or substance use.

Person refers to an employee with signs and symptoms of a common mental health problem.

Mental health first aid means the help provided by any employee to another employee who is developing a mental health problem or experiencing a mental health crisis. The help is provided until professional help is received or until the crisis resolves. This help may be provided by one person or by a range of different people.

Helper refers to any employee who provides mental health first aid as defined above. This helper does not necessarily have a designated role, such as a physical first aid officer has. The helper may be a co-worker of the person, be the person's manager, or in a role less senior to the person.

Workplace refers to a place of employment.

Employee refers to a person working for another person, business or organisation (who could also be working as a contractor or volunteer).

Manager refers to a person responsible for the supervision of one or more employees.

Appropriate Manager refers to the person's supervisor, HR, or the business owner depending on the size and nature of the workplace.

Workplace stressors refer to working conditions that can contribute to mental health problems, e.g., long working hours, excessive workload, poor supervisory support.

Reasonable adjustments refers to changes to the work environment that allow a person with a mental illness to work safely and productively.

Disclosure refers to the degree of information being shared with others at work about a person's mental health problem, e.g. functional limitations through to diagnosis.

Employee Assistance Programs refer to a free and confidential counselling service offered by employers to their employees to support their wellbeing in the workplace and in their personal lives.

Workplace Delphi Round 3

3. Approach

There are already guidelines to help people who may be experiencing a range of mental health problems (click here to access these guidelines). We do not wish to replicate existing MHFA guidelines. Rather, our aim is to develop guidelines on how to tailor mental health first aid for a person assisting a co-worker who is experiencing mental health problem in the workplace.

Please rate how important (from 'essential' to 'should not be included') you think it is that each statement be included in the guidelines.

Please keep the definitions in mind when answering the questions.

Approaching the person

* 1. The helper should approach the person showing signs of a mental health problem, even if the person is still working productively.

Essential	Important	Don't know/Depends	Unimportant	Should not be included
◯	◯	◯	◯	◯

* 2. If there is no one else available or appropriate to help, the helper should talk about the situation with someone else who can advise on how to approach the person, while keeping the person's identity anonymous.

Essential	Important	Don't know/ Depends	Unimportant	Should not be included
◯	◯	◯	◯	◯

* 3. If the person is in a role that is senior to the helper and they do not feel they can make the approach, they should consider:

	Essential	Important	Don't know/ Depends	Unimportant	Should not be included
Contacting an impartial, external source of support (e.g. Employee Assistance Program, helpline) for advice	◯	◯	◯	◯	◯
Identifying an appropriate helper within the organisation and contacting them.	◯	◯	◯	◯	◯

Planning the approach

* 4. Before the helper approaches the person, they should be clear about how the concerning behaviours are affecting them.

Essential	Important	Don't know/Depends	Unimportant	Should not be included
◯	◯	◯	◯	◯

* 5. The helper should consider approaching the person by having a casual conversation and asking whether they are OK.

Essential	Important	Don't know/Depends	Unimportant	Should not be included
◯	◯	◯	◯	◯

* 6. When approaching the person, the helper should consider a staggered approach, e.g. starting with a casual approach asking how they are, followed by a meeting if appropriate.

Essential	Important	Don't know/Depends	Unimportant	Should not be included
◯	◯	◯	◯	◯

How the person's MANAGER would make the approach

* 7. Before approaching the person, the MANAGER should:

	Essential	Important	Don't know/ Depends	Unimportant	Should not be included
Consider the expected outcomes and plan the structure and aims of the discussion in advance.	○	○	○	○	○
Be clear about how the concerning behaviours are affecting others.	○	○	○	○	○

* 8. If the MANAGER wishes to approach a person who works offsite, they should organise the visit ahead of time with the person, rather than doing it unannounced.

Essential	Important	Don't know/Depends	Unimportant	Should not be included
○	○	○	○	○

* 9. When talking with the person about their concerns, the MANAGER should:

	Essential	Important	Don't know/ Depends	Unimportant	Should not be included
Focus the discussion on the person's health and wellbeing, rather than their work performance.	○	○	○	○	○
Be honest and open when the person's performance is being affected, even if they are worried that this may distress the person.	○	○	○	○	○

* 10. After raising their concerns with the person, the MANAGER should ask:

	Essential	Important	Don't know/ Depends	Unimportant	Should not be included
What impact any mental health problems are having on them	○	○	○	○	○
Whether any non-workplace issues are contributing to their mental health problems.	○	○	○	○	○

Workplace Delphi Round 3

4. Communication, Support and Disclosure

There are already guidelines to help people who may be experiencing a range of mental health problems (click here to access these guidelines). We do not wish to replicate existing MHFA guidelines. Rather, our aim is to develop guidelines on how to tailor mental health first aid for a person assisting a co-worker who is experiencing mental health problem in the workplace.

Please rate how important (from ‘essential’ to ‘should not be included’) you think it is that each statement be included in the guidelines.

Please keep the definitions in mind when answering the questions.

Communication and Support

* 11. The helper should ask the person whether any non-workplace issues are contributing to their mental health problems.

Essential	Important	Don't know/ Depends	Unimportant	Should not be included
○	○	○	○	○

* 12. If the person says that workplace stressors are contributing to their mental health problem, the helper should encourage the person to talk to an appropriate manager about this.

Essential	Important	Don't know/ Depends	Unimportant	Should not be included
○	○	○	○	○

* 13. The helper should **not** start the conversation with the person by talking about how their own personal struggles have affected their work behaviour.

Essential	Important	Don't know/Depends	Unimportant	Should not be included
○	○	○	○	○

* 14. If the person chooses not to talk, the helper should provide information for the person to take away and look at later.

Essential	Important	Don't know/ Depends	Unimportant	Should not be included
○	○	○	○	○

* 15. If the person is distressed, the helper should do the following (where appropriate to the circumstances and the helper's work role) ring the EAP (if available), with the person's permission, and let the person speak to them.

Essential	Important	Don't know/ Depends	Unimportant	Should not be included
○	○	○	○	○

* 16. If the person has an advance directive or management plan, the helper should seek assistance from an appropriate manager in order to follow it.

Essential	Important	Don't know/ Depends	Unimportant	Should not be included
○	○	○	○	○

Note: The following items relate to how the person's MANAGER would provide support to the person.

* 17. If the person has chosen **not** to disclose their mental health issues to co-workers, the MANAGER should only speak with co-workers about the person in general terms without breaching confidentiality.

Essential	Important	Don't know/Depends	Unimportant	Should not be included
○	○	○	○	○

* 18. The MANAGER should ask the person's permission to encourage co-workers to support the person, whilst still respecting the person's privacy.

Essential	Important	Don't know/Depends	Unimportant	Should not be included
○	○	○	○	○

* 19. The MANAGER should assist the person to identify workplace triggers that contribute to their mental health problems.

Essential	Important	Don't know/Depends	Unimportant	Should not be included
○	○	○	○	○

Disclosure

* 20. *If the person discloses that they have a mental health problem,* the helper should ask them:

	Essential	Important	Don't know/ Depends	Unimportant	Should not be included
How long they have been experiencing a mental health problem, to determine whether this is an ongoing issue or something where an immediate action could assist the situation	○	○	○	○	○
If they have an advance directive or management plan in the workplace in case of an episode, if the person discloses a diagnosis of a mental illness	○	○	○	○	○
If there are any non-work related issues they may like to talk about or make the helper aware of.	○	○	○	○	○

Workplace Delphi Round 3

5. Substance Use, Performance and Reasonable Adjustments

There are already guidelines to help people who may be experiencing a range of mental health problems (click here to access these guidelines). We do not wish to replicate existing MHFA guidelines. Rather, our aim is to develop guidelines on how to tailor mental health first aid for a person assisting a co-worker who is experiencing mental health problem in the workplace.

Please rate how important (from ‘essential’ to ‘should not be included’) you think it is that each statement be included in the guidelines.

Please keep the definitions in mind when answering the questions.

Substance Use Problems and Intoxication

* 21. If the person appears to be affected by drugs or alcohol while at work, the helper should notify an appropriate authority or emergency contact if the person insists on driving home.

Essential	Important	Don't know/ Depends	Unimportant	Should not be included
○	○	○	○	○

* 22. If the person appears to be affected by drugs or alcohol while at work, the MANAGER should:

	Essential	Important	Don't know/ Depends	Unimportant	Should not be included
Arrange for the person to be escorted home safely.	○	○	○	○	○
Notify an appropriate authority or emergency contact if the person insists on driving home.	○	○	○	○	○

* 23. The helper should **not** organise a team meeting to confront the person, even if all other attempts to help the person who is misusing substances fail.

Essential	Important	Don't know/Depends	Unimportant	Should not be included
○	○	○	○	○

Performance Management

* 24. The MANAGER should focus on providing appropriate support for the person's mental health problems, before they address any performance issues.

Essential	Important	Don't know/Depends	Unimportant	Should not be included
○	○	○	○	○

* 25. If at any point it becomes apparent that the person's behaviours are breaching the workplace's rules or regulations, the MANAGER should end the conversation and explain that a formal meeting will need to be arranged.

Essential	Important	Don't know/Depends	Unimportant	Should not be included
○	○	○	○	○

Reasonable Adjustments

* 26. The helper should be able to explain the concept of 'reasonable adjustments' to the person.

Essential	Important	Don't know/Depends	Unimportant	Should not be included
○	○	○	○	○

Workplace Delphi Round 3

6. End of Survey

Thank you for your time!

That is the end of the Round 3 survey. Thank you very much for your contribution to this research.

If participating in this survey has caused you distress then we advise you to talk to a supportive person about these feelings. You might wish to talk to someone using the relevant helpline listed below.

Australia: Lifeline on 13 11 14

New Zealand: Lifeline Aotearoa on 0800 543 354

UK: Samaritans on 08457 909090

Republic of Ireland: Samaritans on 116 123

USA: National Suicide prevention Lifeline on 1800 273 TALK (8255)

Canada: National Suicide prevention Lifeline on 1800 273 TALK (8255)

By pressing the *'Next'* button your responses will be registered with our survey software. Once all panel members have lodged their responses, we will collate the data and send you a report on the final findings.
