# Supplementary material for: Providing mental health first aid in the workplace: a Delphi consensus study
Source: BMC Psychol. 2016 Aug 2;4:41. doi: 10.1186/s40359-016-0148-x (PMC4971664; doi:10.1186/s40359-016-0148-x)
Supplement: Additional file 3: — Final guidelines document. (PDF 1277 kb) [file 40359_2016_148_MOESM3_ESM.pdf]

PROVIDING MENTAL HEALTH FIRST AID TO A CO-WORKER

MENTAL HEALTH FIRST AID GUIDELINES

Mental health problems are common in the workplace and the symptoms can affect performance, e.g. due to problems with concentration, memory, decision-making and motivation. While some people with mental health problems manage their symptoms without impact on their work performance, others may require short-term or ongoing workplace supports. Most people with mental health problems who receive treatment respond with improved work performance. Investing time and support to retain an experienced and skilled employee with a mental health problem is usually more cost-effective than recruiting and training a new person.

Work can be beneficial or harmful to mental health depending on the circumstances. If a person has a mental health problem, being at work in a supportive workplace can assist in their recovery. The level of support needed will fluctuate, as the symptoms of most mental health problems come and go over time.

Providing mental health first aid when a worker is showing the early signs and symptoms of a mental health problem is important, as it can assist the person to return to their usual performance quickly. Failing to provide mental health first aid can lead to the problem worsening or a crisis emerging.

Mental health first aid is defined as *'the help offered to a person developing a mental health problem, experiencing a worsening of an existing mental health problem or a mental health crisis. The first aid is given until appropriate professional help is received or until the crisis resolves'.*

Box 1: Signs and symptoms of a mental health problem at work

Behaviours

- Not getting things done
- Erratic behaviour
- Emotional responses
- Complaints about a lack of management support
- Fixation with fair treatment issues
- Complaints of not coping with workload
- Appearing withdrawn
- Reduced participation in work activities
- Increased consumption of caffeine, alcohol, cigarettes and/or sedatives
- Inability to concentrate
- Being indecisive
- Difficulty with memory
- Loss of confidence
- Unplanned absences
- Conflict with team members/ manager
- Use of grievance procedures
- Increased errors and/or accidents.

Physical/physiological signs

- Tired all the time
- Sick and run down
- Headaches
- Persistent/resistant musculo-skeletal complaints
- Reduced reaction times
- Difficulty sleeping
- Weight loss or gain
- Dishevelled appearance
- Gastro-intestinal disorders.

Source: http://returntowork.workplace-mentalhealth.net.au/

All MHFA guidelines can be downloaded from mhfa.com.au

PROVIDING MENTAL HEALTH FIRST AID TO A CO-WORKER

MENTAL HEALTH FIRST AID GUIDELINES

MENTAL HEALTH FIRST AID

Box 2: How work can contribute to mental health problems

A supportive workplace can help contribute to good mental health in employees. However, a poor quality work environment can increase the risk of developing mental health problems at work. A poor quality work environment may involve some of the following:

- Work demands with little control over tasks, particularly with a lack of support from co-workers
- Work requires high effort, but leads to little rewards (whether it be financial, or few opportunities for advancement)
- Bullying, discrimination and harassment
- Employees being treated unfairly.

Making the approach

If you notice changes in the person's work or interactions with others that may indicate a mental health problem, you should discuss these with the person. It is important to approach the person, whether or not work is a contributing factor.

There are factors other than mental health problems that contribute to problem behaviours in the workplace, e.g. poor performance, interpersonal conflict. Therefore consider whether the problems you have observed may be due to other factors, e.g. workplace culture or interpersonal dynamics rather than mental health problems. If you are aware that the person has a history of mental health problems, you should not assume that their moods, behaviours or any poor performance are necessarily due to these problems.

Deciding whether to approach

You should consider the following in deciding whether you are the right person to approach the person:

- Whether you have the appropriate knowledge and skills to assist the person
- Whether you have any negative experiences, attitudes or beliefs towards people with mental health problems
- Whether you are part of a workplace culture that is contributing to the person's mental health problem.

If you are the person's manager, when deciding whether or not to approach the person, consider whether you may be contributing to the person's mental health problem and if there is a conflict between your management and first aider roles.

If after considering the above, you think that it is not appropriate for you to approach the person, consider talking about the situation with someone who may be in a better position to help and ask them to approach the person. If you seek advice from someone else, you should emphasise the importance of maintaining the person's privacy and confidentiality.

If the person is in a role that is senior to you and you do not feel you can make the approach, you should consider contacting an impartial, external source of support, e.g. Employee Assistance Program (EAP), helpline for advice.

If the person's behaviour is affecting others or posing a risk to health and safety

If the person's behaviour is affecting others in the workplace and the person's manager has appropriate training, you should approach this manager. See Box 3 on what is appropriate training.

If the person's mental health problem is posing a risk to the health and safety of themselves or others in the workplace (e.g. using substances at work), document your concerns, including time, date and location of the concerning behaviours. Talk to an appropriate manager about the concerning behaviours.

Planning your approach

Before approaching the person:

- Make a plan for what you want to say.
- Be clear in your own mind about concerning behaviours you have noticed.
- Find out what resources are available in the workplace to support people with mental health problems, e.g. EAP.
- Be familiar with relevant laws and organisational policies and procedures, such as accommodating employees with mental health problems and anti-discrimination.
- Be familiar with the guidelines on how to help someone who is suicidal (http://bit.ly/MHFASuicide).

Picking a suitable time and place

Think about a time and place to meet that best suits you and the person, e.g. taking into account if the person seems better at the beginning or end of the day. You should have the conversation in an appropriate neutral private space. Ensure the meeting place is free of distractions and interruptions, e.g. switch off mobile phones. Consider approaching the person by having a casual conversation and asking whether they are okay. Allow sufficient time for discussion with the person.

Box 3: What is appropriate training?

Appropriate mental health training for employees should include information about:

- Mental health problems
- Mental health problems and the workplace
- The importance of early identification, help-seeking and available support services
- How to watch out for and approach co-workers when you are concerned about their mental health
- Employee rights and responsibilities.

Managers should also receive training in:

- Management styles and practices that promote good mental health
- What to do if an employee refuses to seek help
- How to deal with under-performance issues when mental health problems are involved
- How to take action when an employee reports a mental health problem that poses a health and safety risk to others.

Adapted from Guidelines for Workplace Prevention of Mental Health Problems http://prevention.workplace-mentalhealth.net.au/

PROVIDING MENTAL HEALTH FIRST AID TO A CO-WORKER

MENTAL HEALTH FIRST AID GUIDELINES

Additional considerations if you are the person's manager

Before approaching the person, document any concerning behaviours that you have observed. Consider the expected outcomes and plan the structure and aims of the discussion in advance. Approach the person directly and privately about your concerns, rather than indirectly with a group of employees, e.g. holding a meeting to talk about general concerns about 'some employees' or running training.

Listening and communicating non-judgmentally

Confidentiality

You should maintain the person's confidentiality and privacy except where there is a concern for the safety of the person or others. For example, even within a management team, it is not okay to tell a fellow manager about an employee's mental health unless consent has been given.

Be clear with the person about the limits of confidentiality. Do this early in your conversation. Discuss and agree with the person about any limits on confidentiality and who will be told what, e.g. when needing advice from Workplace Health & Safety (WHS), Human Resources or others.

Building trust

It is important to create a supportive and trusting environment when discussing mental health problems with the person. There are several ways to do this. Sit an appropriate distance away, taking into account personal space. Consider what you know about the person and their culture. Engage with the person by using culturally appropriate eye contact.

Ask open-ended questions about how any mental health problems may be affecting the person. Allow the person time to talk, listen non-judgmentally and demonstrate empathy and respect.

You don't need to have all the answers; it's more important to be motivated by care and concern than to say the 'right things'. Stop and try again if what you say doesn't sound quite right, as it doesn't have to be the end of the conversation. Listen if the person is willing to discuss any mental health problems. If the person has opened up and shared their story, you should express your appreciation.

When talking with the person

When talking with the person, you should describe the specific observations and reasons for your concern. Express your concerns in a non-confrontational and clear manner, using examples of what you feel are the concerning changes. State changes you have observed in a positive way, e.g. "I have noticed that you are not your usual cheery self" or "you seem less energetic". Ask questions in an open and exploratory way, e.g. "I've noticed that you've been arriving late recently and wondered if there was a problem?" After raising your concerns with the person, ask whether they would feel more comfortable discussing the problem with someone else.

Be aware that there may be pros and cons for the person in disclosing any mental health problems at work, see Box 4. If the person discloses that they have a mental health problem, you should ask them what impact the mental health problem is having on them. You could also ask them if there are any non-work related issues they may like to talk about or make you aware of. If you are unsure about what terminology to use when talking with the person about their mental health problem, ask the person what terms they use themselves.

Ask the person whether any workplace stressors or other issues are contributing to their mental health problem. Be aware that not all workplace stressors affect everyone in the same way. If the person says that workplace stressors *are* contributing to their mental health problem, encourage the person to talk to an appropriate manager about this.

Box 4: Pros and cons to disclosing a mental health problem at work

Disclosing a mental health problem at work involves a person sharing information with a co-worker about their experience of the mental health problem. A person may choose to disclose for various reasons like requiring support or to explain behaviour, such as during ill-health or performance management processes. They may choose to share only some information with one person, such as the presence of a diagnosis and the need for adjustments to their manager. Or they may feel they can speak openly about their experiences with many co-workers. The level of disclosure will vary according to the person's circumstances with their mental health and their employment. There are a number of benefits and possible risks that a person will weigh up when deciding whether to disclose, which are summarised below.

Pros to disclosing

- Enables reasonable adjustments to be made
- Enables support
- Educates others about mental health problems
- Reduces stress associated with concealing mental health problem
- Explains behaviours associated with mental health problem that may have been misinterpreted or mislabelled
- Can lead to positive experiences with co-workers, such as others disclosing
- Means person can be honest about their mental health problem.

Cons to disclosing

- Could lead to unfair treatment/ discrimination like reduced likelihood of promotion or job loss
- Employer may not provide appropriate support
- Could promote gossip
- Could lead to rejection or ostracism by co-workers
- Could lead to co-workers devaluing or undermining the person.

Note there may be reasons why a person may not wish to disclose such as them not requiring workplace adjustments or support or not wishing to share personal information with co-workers.

Adapted from Brohan, et al. BMC Psychiatry 2012.

PROVIDING MENTAL HEALTH FIRST AID TO A CO-WORKER

MENTAL HEALTH FIRST AID GUIDELINES

You should offer to assist them to find appropriate information and support.

At the end of the conversation, you should agree with the person what will happen next and who will take action. If after the conversation you feel distressed, you should find someone to talk to for support and advice, while respecting the person's privacy. If your expectations of the discussion are not met, be aware that your actions may still make a difference, e.g. the person may speak to someone else about their mental health problem.

Don'ts

You should not:

- Start the conversation with the person by talking about how your own personal struggles have affected your work behaviour
- Make the person talk about their mental health problem if they don't want to
- Ask questions that create pressure like "What's wrong with you?" or "Are you stressed or something?"
- Rush in with another question without listening to the answer
- Diagnose the person with a mental illness or use diagnostic terminology unless the person uses it
- Try to act as a counsellor, tell the person what to do, or offer the person remedies or treatments
- Tell the person that they need to stay busy, get out more, 'snap out of it' or to 'get your act together'
- Be patronising, blame, accuse, treat the person as an invalid or embarrass the person by saying things like 'everyone is noticing…'
- Assume the person's problems will just go away
- Minimise the person's problems by comparing them to your own problems or those of other people
- Say "I've had the same experience" unless you have
- Make assumptions about whether any sickness absence of the person is 'genuine'
- Minimise the problems the person may encounter as a result of their mental health problems.

Box 5: Talking with co-workers about the person

When you are talking with another employee about your concerns about the person, you should be specific about your observations and avoid critical comments, e.g. "I have seen Lin engaged in heated arguments with two co-workers and even a client in the past week" rather than "Lin has gone crazy". Ensure you use non-stigmatising language when talking about the person, e.g.'people with schizophrenia' rather than 'schizophrenics', 'people with substance use problems' rather than 'addicts'. Be careful not to label the person's mental health problems as 'bad' behaviour, e.g. referring to a person who is frequently late as 'lazy'.

When you are talking with another employee who is concerned about the person, focus on possible solutions or support rather than solely on the problem. Do not complain to other employees or spread rumours about the person. If you hear other employees talking about the details of the person's mental health problems in an unhelpful way, let them know it is inappropriate. You should also let an appropriate manager know.

What if I'm not the right person to help?

If while talking with the person, you decide that you are not the right person to help, tell them this and discuss alternative sources of help. You can also offer to seek the immediate assistance of someone with appropriate training, e.g. a Mental Health First Aid Officer or the EAP.

What if they don't want to talk?

Be prepared for the possibility that the person may not accept your support. Remain calm if the person reacts in a negative way, e.g. denial, anger. Try not to take it personally if they don't want to talk.

Be aware that the person may be reluctant to talk about any mental health problems for various reasons, see Box 4 about the pros and cons to disclosure. Respect the person's wishes if they do not wish to discuss non-work related issues.

If the person chooses not to talk, tell them that you are available to talk in the future if they wish to and encourage them to talk to someone else they trust instead. You could also provide information for the person to take away and look at later. You should also touch base with the person at a later time to see if they are more willing to talk.

Additional considerations if you are the person's manager

Ensure you first talk about the person's strengths and how they are valued. Emphasise your concern for the person's wellbeing rather than their work performance and keep this as the focus of the discussion. After raising your concerns with the person, ask if they would like to continue the conversation in the presence of a support person, e.g. an external advocate, co-worker.

If the person has a substance use problem, explain the effects that their substance use could have on co-workers and the workplace. Point out the possible workplace consequences of substance use, e.g. loss of job if working while intoxicated.

Giving support and information

Ask the person what support they need right now, but be aware they may not be able to think clearly about solutions. If this is the case, assist them in exploring the available options. Offer information about available support and encourage the person to seek appropriate professional help. Be aware of the barriers to help-seeking for a person with a mental health problem, e.g. embarrassment, shame or believing they should be able to cope on their own.

Encourage the person to use any helpful coping strategies they have used in the past. If the person has a relapse management plan, follow the

instructions. Have a discussion about how the workplace can support the person's existing coping strategies. Make sure the person is aware of any relevant supports that the organisation can provide, e.g. the EAP. You should also ask about supports outside of work that the person may find helpful.

If the person is having difficulties with their performance because of their mental health problem, suggest they speak to an appropriate manager about reasonable adjustments. You should be able to explain the concept of 'reasonable adjustments' to the person (see Box 6).

If it's okay with the person, check in with them regularly in order to provide ongoing support and encouragement. You can also informally check in with the person to see how they are doing.

Box 6: What are reasonable adjustments?

Reasonable adjustments refer to changes to the work environment that allow a person with a mental illness to work safely and productively. Examples of adjustments an employer may need to make are reviewing and adjusting the performance requirements of the job or arranging flexible work hours. Many employees with mental health problems will not need any adjustments, and some may only require minor changes.

Additional considerations if you are the person's manager

Be aware of relevant guidelines on how to carry out reasonable adjustments for the person. If the organisation has no policies for accommodating employees with mental health problems, you should consider the best way to accommodate the person in their role. You may wish to seek advice from an appropriate source, e.g. Human Resources, EAP. Be aware of any legal obligations that the person has to disclose that they have a mental health problem at work, e.g. medical practitioners may be required to report medical conditions that impair their performance.

Ask the person what support they need in order to fulfil the inherent requirements of their job. Tell the person that you will help them get the support they need that will assist them in returning to their usual work performance. Assist the person to identify workplace triggers that contribute to their mental health problem. If the person is having difficulties with their performance because of their mental health problem, you should discuss reasonable adjustments with them. Should the person not wish to disclose their mental health problem to co-workers, discuss what adjustments are possible without making staff aware that the person has a mental health problem. Be aware of the possible impact of the person's mental health problem on other employees and offer support to them as well.

Discuss with the person what information they would like their co-workers to be given or need to be given about the person's mental health problem. If the person has chosen not to disclose their mental health problem to co-workers, you should only speak with co-workers about the person in general terms without breaching confidentiality. Tell the person that you will not tolerate any discrimination towards or harassment of them at work due to their mental health problem.

Consider having regular discussions with the person to monitor how they are coping with their workload and workplace accommodations. Provide the person with an opportunity to talk to someone, for when they might be feeling distressed at work, e.g. telephone helpline. Try to adapt the way you manage the person in order to reduce any work-related stress, e.g. some people may function better with more direction from their manager.

Managing performance issues

Focus on providing appropriate support for the person's mental health problem, before you address any performance issues. Be aware that behaviours that breach the workplace's rules or regulations may require both performance management and mental health first aid.

If there are any performance or behaviour issues, explain clearly what is expected of the person in terms of work performance and behaviour. When you are providing support for the person's mental health problems and are required to initiate performance management processes, you should be specific about the purpose of any meeting. You should also ask the person if they would like a support person present.

Helping the distressed co-worker

If the person is distressed, do not ignore them, as doing nothing can make the situation worse. Where appropriate to the circumstances and your work role, consider doing the following:

- Provide an appropriate space where the person can express emotion freely and compose themselves in privacy.
- Assess for any crisis (immediate risk of harm to themselves or others) and respond according to existing guidelines for suicidal thoughts and behaviours (http://bit.ly/MHFASuicide).
- Acknowledge the person's distress, and focus on what is distressing them right now, rather than immediately try to problem solve.
- Reassure them that it is okay to be upset, and that they are valued; they may be feeling embarrassed or ashamed about what happened.
- Let the person know you are listening; they may be comforted by just having someone there to listen to them.
- Tell them that help and support will be offered.
- Don't feel you need to offer the person an immediate solution, especially if it has long-term implications.
- Ask the person if they need you to contact someone on their behalf.

- Let them know that once they have recovered sufficiently, it is okay to carry on working, take a break, or seek permission to go home.

Be aware that the person may not be able to think clearly and take in much information. If they are too distressed to talk, reassure the person that the discussion can continue at another time and place that suits them. If needed, seek assistance to more effectively support a person who is distressed, while maintaining the person's privacy. Ring the EAP (if available), with the person's permission, and let the person speak to them.

If needed, seek support for yourself after assisting a person who is distressed, while maintaining the person's confidentiality, for example making use of EAP services.

Helping the intoxicated co-worker

If the person appears to be affected by alcohol or other drugs while at work, you should speak to an appropriate manager. Do not ignore this or cover for them, e.g. by carrying out their work tasks. Try to ensure that the person does not pose a health and safety risk to themselves or others. Escort the person to a private area and inform them of your concerns. If the person insists on driving home, notify an appropriate authority or emergency contact.

Additional considerations if you are the person's manager

You should also ask the person for any explanation for their behaviour, e.g. side effects of a medication. Arrange for the person to be escorted home safely.

Purpose of these guidelines

These guidelines describe how members of the public should tailor their approach when providing mental health first aid to a co-worker who may be developing a mental health problem, experiencing a worsening of an existing mental health problem or in a mental health crisis. The role of the mental health first aider is to assist the co-worker until appropriate professional help is received or the crisis resolves.

Development of these guidelines

These guidelines are based on the expert opinions of people with lived experience of mental health problems whilst working, managers with experience supervising employees with mental health problems and workplace mental health professionals. The experts were from Australia, Ireland, New Zealand, the UK and the USA.

How to use these guidelines

These guidelines are a general set of recommendations. Each individual is unique and it is important to tailor your support to that person's needs. Therefore, these recommendations may not be appropriate for every person. Also, the guidelines are designed to be suitable for providing first aid in developed English-speaking countries. They may not be suitable for other cultural groups or for countries with different health systems.

These guidelines have been developed as part of a suite of guidelines about how to best assist a person with mental health problems. These other guidelines can be downloaded at:

mhfa.com.au/resources/mental-health-first-aid-guidelines

Although these guidelines are copyright, they can be freely reproduced for non-profit purposes provided the source is acknowledged. Please cite the guidelines as follows:

Mental Health First Aid Australia. Providing mental health first aid to a co-worker. Melbourne: Mental Health First Aid Australia; 2016.

Enquiries should be sent to: Mental Health First Aid Australia
email: mhfa@mhfa.com.au
